# On the predictability of event boundaries in discourse: An ERP investigation

**DOI:** 10.3758/s13421-017-0766-4

**Published:** 2017-11-20

**Authors:** Francesca Delogu, Heiner Drenhaus, Matthew W. Crocker

**Affiliations:** Department of Language Science & Technology, Building C7.1, Saarland University, 66123 Saarbruecken, Germany

**Keywords:** Situation models, Event boundaries, Event Segmentation Theory, Model updating, ERPs

## Abstract

When reading a text describing an everyday activity, comprehenders build a model of the situation described that includes prior knowledge of the entities, locations, and sequences of actions that typically occur within the event. Previous work has demonstrated that such knowledge guides the processing of incoming information by making event boundaries more or less expected. In the present ERP study, we investigated whether comprehenders’ expectations about event boundaries are influenced by how elaborately common events are described in the context. Participants read short stories in which a common activity (e.g., washing the dishes) was described either in brief or in an elaborate manner. The final sentence contained a target word referring to a more predictable action marking a fine event boundary (e.g., drying) or a less predictable action, marking a coarse event boundary (e.g., jogging). The results revealed a larger N400 effect for coarse event boundaries compared to fine event boundaries, but no interaction with description length. Between 600 and 1000 ms, however, elaborate contexts elicited a larger frontal positivity compared to brief contexts. This effect was largely driven by less predictable targets, marking coarse event boundaries. We interpret the P600 effect as indexing the updating of the situation model at event boundaries, consistent with Event Segmentation Theory (EST). The updating process is more demanding with coarse event boundaries, which presumably require the construction of a new situation model.

**Electronic supplementary material:**

The online version of this article (10.3758/s13421-017-0766-4) contains supplementary material, which is available to authorized users.

## Introduction

A great deal of research indicates that knowledge about real-world events or everyday activities, also called *event schemata* or *script knowledge* (Schank & Abelson, 1977), is rapidly activated and influences online processing at the level of individual words (e.g., Chwilla & Kolk, 2005; Hare, Jones, Thomson, Kelly, & McRae, 2009; McRae, Hare, Elman, & Ferretti, 2005), sentences (e.g., Altmann & Kamide, 1999; Bicknell, Elman, Hare, McRae, & Kutas, 2010; Matsuki et al., 2011), and wider discourse (e.g., Camblin, Gordon, & Swaab, 2007; Metusalem, Kutas, Hare, McRae, & Elman, 2012; Otten & van Berkum, 2007). In particular, research on discourse comprehension has shown that event knowledge plays a crucial role in building incremental representations of the situation described in a text, so-called *mental* or *situation models* (Johnson-Laird, 1983; Van Dijk & Kintsch, 1983; Zwaan & Radvansky, 1998). Situation models arise through the integration of information explicitly presented in the text with reader’s prior knowledge about the participants, locations, and sequences of actions that typically occur within the event described (e.g., Kintsch & van Dijk, 1978). Incoming information is evaluated against the current state of the situation model: When it is consistent with it, it is integrated and the current representation remains active; when a topic shift, or a change in character, location, time, or activity signals an event boundary, the current model is updated, and/or a new model initiated (Bestgen & Vonk, 2000; Gernsbacher, 1990). A number of studies provide evidence that updating situation models involves extra processing operations, as evidenced by increased reading times (e.g., Bestgen & Vonk, 2000; Rinck & Weber, 2003; Speer & Zacks, 2005; Zacks, Speer, & Reynolds, 2009; Zwaan, 1996; Zwaan, Magliano, & Graesser, 1995) and neural activity (Speer, Reynolds, Swallow, & Zacks, 2009; Speer, Zacks, & Reynolds, 2007; Whitney et al., 2009).

Speer et al., (2007), for example, presented participants with narrative texts about everyday events while brain activity was recorded with functional magnetic resonance imaging (fMRI). Participants subsequently segmented the text into sub-events at a fine-grained level (i.e., marking the smallest units of activity that seemed natural and meaningful) or at a coarse-grained level (i.e., marking the largest units of activity that seemed natural and meaningful). The results revealed increased neural activity in a network of brain regions at the points that were later identified as boundaries between events. Interestingly, in the majority of these regions, larger responses were evoked by coarse-grained than fine-grained boundaries, suggesting that brain activity was modulated by the hierarchical structure of the events. This sensitivity to event structure has also been observed when people view videos of everyday activities (Zacks et al., 2001; Zacks & Swallow, 2007).

These findings indicate that the effort required to update a situation model is sensitive to the structure of events. However, why should updating at coarse event boundaries be more effortful than it is at fine event boundaries? One possible answer to this question is provided by Event Segmentation Theory (EST; Zacks, Speer, Swallow, Braver, & Reynolds, 2007), an approach originally developed for event perception but extended to account for narrative comprehension as well (Zacks et al., 2009). According to EST, comprehenders use their knowledge of events to predict what might happen next. Most of the time, these predictions turn out to be accurate. At event boundaries, however, future activity becomes less predictable, causing an increase in prediction error. Higher levels of prediction error trigger the reset and updating of the current situation model. Zacks (2010) describes these mechanisms using the following example: if you are watching a person putting on a pair of shoes, you can use your representation of the shoe-tying event to predict that after the first shoe has been tied, the person will move to the second one. Once both shoes have been tied, however, this event representation will no longer be helpful to generate accurate predictions, causing levels of prediction error to increase. At this point the system triggers an updating process in which a new set of event representations is formed and an event boundary perceived. Hence, the processing difference between fine and coarse event boundaries may simply reflect a difference in levels of prediction error. Fine event boundaries mark predictable events continuing the current activity (in a chronological sequence), and are therefore associated with lower levels of prediction error; coarse event boundaries, by contrast, are associated with higher levels of prediction error as they initiate new, unpredictable events. EST predicts that coarse event boundaries will trigger a global update of the current situation model, resulting in a processing cost. At fine event boundaries, by contrasts, the current situation model is maintained, or, presumably, only updated with respect to the individual incoming event (see the difference between incremental and global updating postulated by e.g., Kurby & Zacks, 2012).

If this hypothesis correctly characterizes processing in real-time, incoming events marking coarse event boundaries (i.e., events initiating less predictable, new activity) should be more difficult to process than incoming events marking fine event boundaries (i.e., more predictable events, continuing the current activity). For example, a situation model representing a washing-the-dishes scenario should be easier to update with a drying-the-dishes event than with a jogging event because, based on script knowledge about washing-the-dishes activities, drying the dishes is more predictable than jogging (cf. findings from Sitnikova, Holcomb, Kiyonaga, & Kuperberg, 2008, in the context of filmed events). Integrating a more predictable event involves maintaining the current situation model and updating it with the individual dimension that changed (which, in turn, leads to interpret the incoming event as part of the ongoing episode). On the other hand, a less predictable jogging event requires a more demanding global updating process, in which the old model is reset and a new one constructed. This should lead the incoming event to be interpreted as part of a new story episode (Bailey & Zacks, 2015; Kurby & Zacks, 2012; Magliano, Miller, & Zwaan, 2001).

Beyond this quite uncontroversial prediction, the present study further examined whether comprehenders’ expectations for fine vs. coarse event boundaries are modulated by how elaborately the scenario is described in the context. A washing-the-dishes activity can be described by introducing just one salient action (e.g., washing the plates) or multiple typical actions of the superordinate (dish-washing) activity (e.g., washing cups, cutlery and plates), and this may in turn influence the degree to which incoming information is expected to mark a fine or a coarse event boundary. We expect that, relative to a *brief* context describing a single action, an *elaborate* context—introducing an event involving multiple actions—should lead to higher expectations that the activity is completed, thereby triggering the updating of the current situation model. This is because, while script knowledge about the ongoing activity is still relevant to generate expectations for subordinate actions in brief contexts (e.g., we know that washing the dishes involves washing plates, but also cups, cutlery, etc.), it contributes less to shape expectations following elaborate contexts, where the ongoing activity has been described at greater length. Notice that our distinction between brief and elaborate contexts parallels the one outlined by Zacks (2010) between the perception of a partial event (when only one shoe has been tied) vs. a complete event (when both shoes have been tied). In a similar vein, elaborate contexts should be associated with higher uncertainty about what might happen next, anticipating that a new event representation will need to be constructed as soon as incoming information is encountered.

We tested these hypotheses in an event-related potential (ERP) study manipulating two factors: The length of the event description in the context (*brief* vs. *elaborate*) and whether the target word referred to a more predictable, related activity (*fine event boundary*) or a less predictable event, initiating new activity (*coarse event boundary*).

We focus on two ERP components that are reported to be modulated during discourse comprehension. The first one is the N400, a negative-going wave peaking approximately 400 ms post stimulus onset, more pronounced over centro-parietal sites. The amplitude of the N400 is sensitive to a variety of factors, including the degree to which a word is expected given its sentential or discourse context (e.g., Federmeier & Kutas, 1999; George, Mannes, & Hoffman, 1997; Otten & van Berkum, 2007; van Berkum, Hagoort, & Brown, 1999, see Kutas & Federmeier, 2011 for a review). For example, Otten and van Berkum (2007) found that highly expected words (e.g., *meeting*) in a supportive context (e.g., *The manager thought that the board of directors should assemble to discuss the issue. He planned a …*) elicit lower N400 amplitudes relative to less expected words (e.g., *session* in the same context). Based on these findings, we expect the N400 elicited by (more predictable) fine event boundaries in the current study to have lower amplitude than that elicited by (less predictable) coarse event boundaries, reflecting easier retrieval processes (e.g., Lau, Phillips, & Poeppel, 2008). Under an integration account of the N400 (e.g., Hagoort, 2003), however, we might also expect an interaction with description length, to the extent that brief contexts provide greater support for fine event boundaries.

The other relevant component is the P600, a positive shift starting at about 500 ms post stimulus onset and lasting for several hundreds of milliseconds. While initially linked to syntactic revision or repair (e.g., Hagoort, Brown, & Groothusen, 1993), P600 effects have been also observed in response to discourse-level processing (see Brouwer, Fitz, & Hoeks, 2012, for a review). In particular, late positivities have been associated with processing of discourse reorganization or discourse model updating and the integration of new referents into the discourse representation (e.g., Brouwer et al., 2012; Burkhardt, 2007; Schumacher & Hung, 2012). In the current study, P600 effects should be seen in response to updating processes like those associated with elaborate contexts. Furthermore, if coarse boundary targets lead to more effortful global updating processes compared to fine boundary targets requiring only incremental updating, we might expect the P600 effect to be stronger at coarse boundary targets.

### Method

#### **Participants**

Twenty-four Saarland University students volunteered to participate in the experiment.^1^ They were all right-handed native German speakers with normal or corrected-to-normal vision and without any history of neurological disorders. All participants provided written informed consent and were paid 15 Euros to take part in the experiment. Four participants were excluded from analysis due to excessive artifacts in the electroencephalogram (EEG).

#### **Materials**

The experimental materials consisted of 120 items in four conditions crossing the factors length (*brief* vs. *elaborate*) of the event description and type of boundary (*fine* vs. *coarse*) marked by the target (see Supplemental Material). An example of the materials is given in Table 1 (see also the Appendix).

**Table 1:** A sample item in each condition (with English translation)

**Intro**
Jörn ist mit dem Frühstück fertig. Er geht in die Küche, ...
*(Jörn has finished breakfast. He goes to the kitchen, ... )*
**Brief event description - Fine event boundary**
... wo er Teller abwäscht. Dann beginnt er mit dem Abtrocknen, *[...]*.
*(... where he washes plates. Then he starts to* dry *, [...])*
**Elaborate event description - Fine event boundary**
...wo er erst Tassen, dann Besteck und dann Teller abwäscht. Dann beginnt er mit dem Abtrocknen, [...]
*(... where he first washes cups, then cutlery and then plates. Then he starts to* dry *, [...])*
**Brief event description - Coarse event boundary**
... wo er Teller abwäscht. Dann beginnt er mit dem Joggen, *[...]*
*(... where he washes plates. Then he starts to* jog, *[...])*
**Elaborate event description - Coarse event boundary**
... wo er erst Tassen, dann Besteck und dann Teller abwäscht. Dann beginnt er mit dem Joggen, *[...]*
*(... where he first washes cups, then cutlery and then plates. Then he starts to* jog, *[...])*

NB: The target word is underlined for illustrative purposes

After a short introductory sentence, the context sentence introduced a common activity by mentioning its typical location and either one typical action (brief event description), or a sequence of actions that typically occur within the activity (elaborate event description). The action in the brief description was always the last action mentioned in the elaborate description, so that immediately prior to the target sentence the same action was mentioned across conditions. The final sentence always started with the expression “Then he starts”, which served as a cue that an event was about to be mentioned. The target word referred to an action that was either naturally continuing the current activity (e.g., drying), thereby marking a fine event boundary, or initiating a new, less predictable activity (e.g., jogging), thereby marking a coarse event boundary.

To ensure that the target words in the fine boundary conditions were perceived as natural continuations of brief as well as elaborate contexts, two groups of 17 students from Saarland University performed cloze norming on these passages. They were presented with one version of each passage pair (brief and elaborate) up to the word preceding the critical word and were asked to fill in the first word that came to mind. The target word in the fine boundary condition was selected in such a way that it appeared as a completion in both types of contexts. The average cloze probability for fine boundary targets was .24 (SD = .18) in brief contexts and .21 (SD = .17) in elaborate contexts. The difference was marginally significant (*t*= 1.96, *p*< 0.06), but it did not produce effects on the N400 amplitude. The events in the coarse boundary condition were chosen to be unrelated to the activity described in the context (for example, by involving a different location than the one mentioned in the context). We also made sure that they were unattested in the cloze completions (cloze probability was 0 in both contexts) so that they were less predictable than the fine boundary targets.

To estimate whether elaborate contexts were indeed more likely to be perceived as describing completed events, and therefore be associated with coarser event boundaries compared to brief contexts, we asked two independent native speakers of German to rate on a scale from 1 to 5 how closely related each completion in the cloze study was to the activity described in the context. For each item and condition, we then computed the percentage of completions rated as more closely (i.e., rated as 1 or 2) vs. less closely (i.e., rated as 3,4, or 5) related to the activity described in the context. The data showed that unrelated completions were indeed more likely to appear following elaborate contexts (36% of completions) than brief contexts (28% of completions). Both raters showed this pattern, with moderate agreement on specific items (Cohen’s kappa = .44, z = 4.75, *p*< 0.0001).

We also assessed the (word-form) frequency of fine and coarse event boundary targets, which was in general very low. Fine event boundary targets occurred on average 5.41 times per million words, while coarse event boundary targets 2.54. The difference was marginally significant (*t*=-1.97, *p*< 0.06). It is well known that, when all other factors are constant, the N400 amplitude is modulated by word frequency (e.g., Rugg, 1990). However, the N400 frequency effect interacts with other factors, including the position of the word in the sentence and its predictability. Van Petten and Kutas (1990), for example, showed that low-frequency words elicit higher N400 amplitudes only when they occur in sentence initial positions. The interaction between word frequency and word position was taken as evidence that the frequency effect can be overridden by contextual constraint (or predictability). Consistent with this, Dambacher, Kliegl, Hofmann, and Jacobs (2006) found interactions of predictability and frequency as well as of position and frequency, with predictability accounting better for N400 effects than word position. Furthermore, they found that the effect of predictability was larger for low-frequency than for high-frequency words (i.e., frequency modulates the strength of the predictability effect on the N400). Since the target words in the present study had low frequency and appeared towards the end of relatively constraining passages, we take any observed N400 modulation to the targets to reflect contextual predictability rather than marginal differences in frequency.

Four counterbalanced lists were created in such a way that each item appeared in only one condition per list, but in all conditions equally often across lists. Within each list, the experimental items were mixed with 120 filler passages of an unrelated experiment. The final sentence of the filler passages always started with a causal or a concessive connector (e.g., *therefore*, *however*) and continued with an event description that was either congruent or incongruent with the expectations driven by the connector type.

#### **Procedure**

Participants were seated in a sound-proof, electro-magnetically shielded chamber. Stimuli were presented with E-prime software (Psychology Software Tools, Inc.) in black fonts on a white background. After a short training session of four trials, the items were presented in pseudo-randomized order, in six blocks with breaks after each block.

Each trial started with a screen prompting participants to press a button to start reading the passages. Each context sentence was presented in its entirety until participants pressed a button. Then a fixation cross appeared for 500 ms, after which the target sentence was presented word-by-word in the center of the screen, for 350 ms plus 100 ms inter-stimulus interval (RSVP).

In 25% of cases, a simple comprehension question requiring a yes/no-answer appeared (e.g., Did Jörn go to the kitchen?). Participants responded by pressing one of two buttons within a maximal interval of 5000 ms. Participants were instructed to read the passages for comprehension and to avoid blinking during the word-by-word presentation of the target sentence.

#### **Electrophysiological recording and processing**

The EEG was recorded by means of 26 Ag/AgCl scalp electrodes placed according to the 10–20 system. The signal was referenced and digitized at a sampling rate of 500 Hz. Data were recorded using FCz as reference and AFz as ground. The horizontal electro-oculogram (EOG) was monitored with two electrodes placed at the outer canthus of each eye and the vertical EOG with two electrodes above and below the right eye. Electrode impedance was kept below 5 kΩ for all scalp electrode sites, and below 10 kΩfor the EOG electrodes. During recording, no on-line filters were used.

The EEG data were band-pass filtered offline with 0.03–30 Hz (slope 12 dB) and re-referenced to the average of the left and right mastoid electrodes. Epochs time-locked to the target words were extracted with an interval of 200 ms preceding and 1200 ms following the onset of the target word and semi-automatically screened for electrode drifts, amplifier blocking, eye and muscle artifacts. Approximately 11% of target word epochs were rejected due to artifacts, with no significant differences between conditions. Averaged ERPs time-locked to the critical word in each condition and for each participant were computed on the artifact-free epochs using a 200 ms pre-stimulus baseline.

### Analyses

Nine representative electrode sites from anterior (F3, Fz, F4), central (C3, Cz, C4), and parietal (P3, Pz, P4) regions were included in the analyses. Based on previous reports and visual inspection of the data, we computed mean amplitudes for each condition and electrode in the 350-550 ms (N400) time window and the 600–1000 ms (P600) time window. Within each time window, we performed ANOVAs with length of event description (two levels: brief, elaborate), type of boundary marked by the target (two levels: fine, coarse), anterior-posterior (AP) distribution (three levels: anterior, central, posterior sites) and lateral distribution (three levels: left, midline, right sites) as within-subject factors. The Greenhouse-Geisser correction was applied to all repeated measures with greater than one degree of freedom in the numerator. In such cases, the corrected *p* value is reported.

### Results

**Fig. 1:**
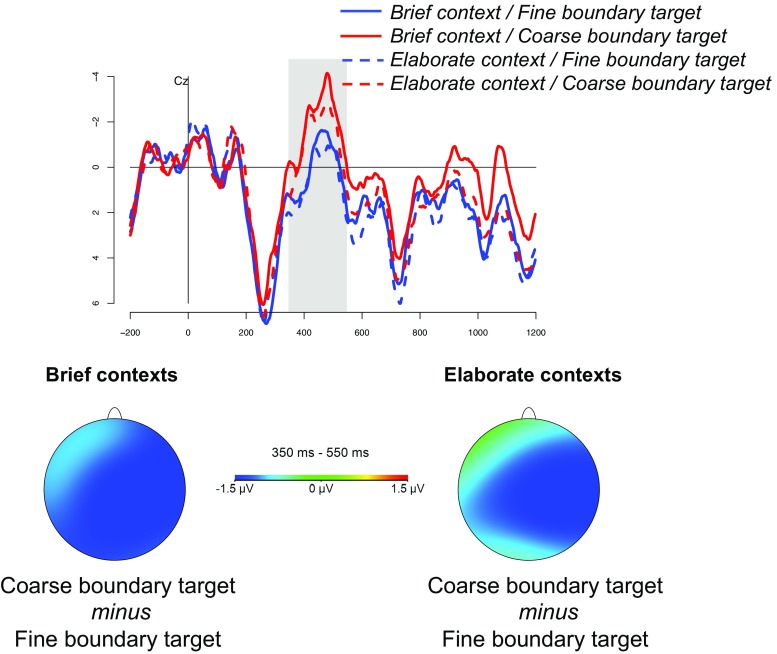
Grand average ERP responses at electrode Cz. The topographic maps show the N400 effect of event boundary in brief and elaborate contexts

**Fig. 2:**
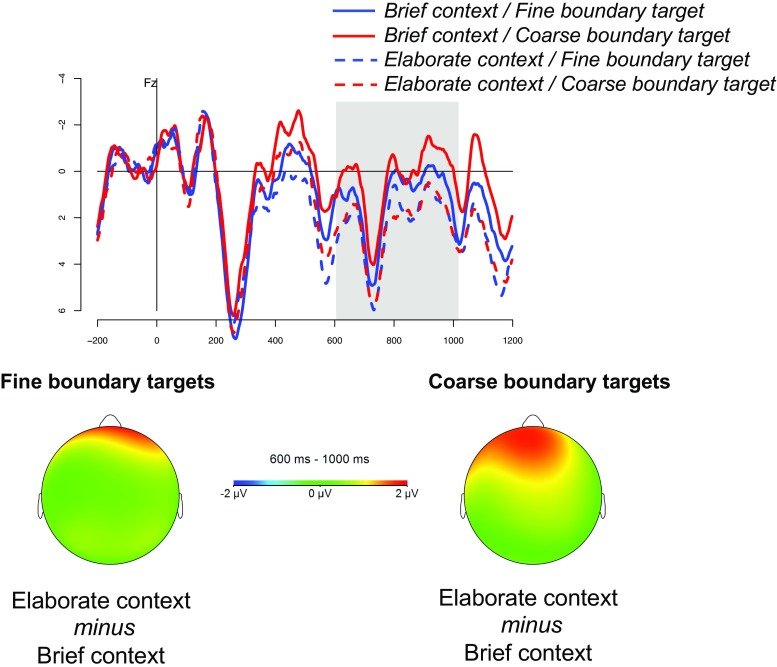
Grand average ERP responses at electrode Fz. The maps show the frontal effect of description length for fine and coarse boundary targets.

On average participants answered over 90% of the questions correctly, indicating that they were engaged in the task. The grand average waveforms at electrodes Cz and Fz are shown in Figs. 1 and 2, respectively. The results of the ANOVAs in the N400 and P600 time windows are reported in Table 2.^2^

**Table 2:** ANOVAs on ERPs to target words across the N400 time window and the P600 time window

Effect	*F (df)*	*p*	${\eta _{p}^{2}}$
*N400 time window: 350-550 ms*			
Boundary	32.90 (1, 19)	.000	.63
Boundary × AP	1.61 (2, 38)	.221	.08
Boundary × Lat	2.55 (2, 38)	.091	.12
Boundary × AP × Lat	1.97 (4, 76)	.137	.09
Length	0.61 (1, 19)	.445	.03
Length × AP	0.94 (2, 38)	.355	.05
Length × Lat	0.07 (2, 38)	.936	< .01
Length × AP × Lat	3.74 (4, 76)	.008	.16
Boundary × Length	0.10 (1, 19)	.761	< .01
Boundary × Length × AP	0.75 (2, 38)	.409	.04
Boundary × Length × Lat	0.30 (2, 38)	.749	.02
Boundary × Length × AP × Lat	1.23 (4, 76)	.307	.06
*P600 time window: 600-1000 ms*			
Boundary	3.16 (1, 19)	.092	.14
Boundary × AP	0.26 (2, 38)	.683	.01
Boundary × Lat	0.39 (2, 38)	.682	.02
Boundary × AP × Lat	1.59 (4, 76)	.208	.08
Length	1.49 (1, 19)	.238	.07
Length × AP	4.66 (2, 38)	.038	.20
Length × Lat	0.55 (2, 38)	.584	.03
Length × AP × Lat	2.92 (4, 76)	.026	.13
Boundary × Length	0.01 (1, 19)	.918	< .01
Boundary × Length × AP	0.52 (2, 38)	.515	.03
Boundary × Length × Lat	1.69 (2, 38)	.207	.08
Boundary × Length × AP × Lat	1.42 (4, 76)	.234	.07

*AP* anterior–posterior distribution, *Lat* lateral distribution

In the N400 time window, coarse boundary targets elicited larger N400 amplitudes (M = -0.48 *μ* V, SD = 3.06) than fine boundary targets (M = 0.78 *μ* V, SD = 2.85). The ANOVA revealed a main effect of boundary type, and no interactions with length or electrode sites (see Table 2). The analysis also revealed a length ×AP × laterality interaction, possibly reflecting the onset of the P600 effect for elaborate descriptions (see below). Follow-up analyses on the relevant subsets of electrodes revealed that this effect was mostly driven by midline electrodes (midline sites: length × AP interaction, *F*(2, 38) = 3.15, *p*< 0.078, ${\eta _{p}^{2}}$ .14).

The analyses in the P600 time window revealed a length × AP interaction. As shown by the topographic maps in Fig. 2, elaborate contexts elicited a larger positivity than brief contexts in anterior sites. The analysis of frontal electrodes showed a significant effect of length (*F*(1,19) = 6.30, *p* = 0.021, ${\eta _{p}^{2}}$ .25), with a larger positivity for elaborate descriptions (M = 1.733 *μ* V, SD = 3.59) compared to brief descriptions (M = 0.625 *μ* V, SD = 4.11). This main effect was qualified by a length ×boundary × laterality interaction (*F*(2,38) = 3.74, *p* = 0.047, ${\eta _{p}^{2}}$ .16). Follow-up comparisons showed a significant main effect of length (*F*(1,19) = 5.66, *p* = 0.028, ${\eta _{p}^{2}}$ .23) and a length × laterality interaction (*F*(2,38) = 10.31, *p*< 0.001, ${\eta _{p}^{2}}$ .35) for coarse boundary targets, but no significant effects for fine boundary targets (*p* s > 0.1).^3^ Central electrodes revealed only a main effect of boundary (*F*(1, 19) = 4.41, *p* = 0.049, ${\eta _{p}^{2}}$ .19), with a larger negativity for coarse boundary targets (M = 1.91 *μ* V, SD = 3.46) compared to fine boundary targets (M = 2.39 *μ* V, SD = 3.24), suggesting that the N400 effect of boundary type was long-lasting in central electrodes. Posterior electrodes did not show any significant effect.

To summarize, the analyses revealed a broadly distributed N400 effect of event boundary type, with continuing events eliciting a reduced N400 amplitude compared to events initiating new activity. In addition, frontal electrodes showed a positive shift that was sensitive to the length of the event description and the type of boundary marked by the target. In particular, elaborate contexts elicited a larger positivity compared to brief contexts. This effect was largely driven by target events initiating new activity: the difference between elaborate and brief contexts was significant for coarse boundary targets, while it did not reach significance for fine boundary targets.

## Discussion

The goal of this ERP study was to examine the processing of event boundaries during online language comprehension. We tested short narratives in which the context introduced an everyday activity by mentioning just one salient event vs. multiple typical events of the superordinate activity. The final sentence contained a target word referring either to an event that could be expected to continue the current activity (i.e., marking a fine event boundary) or to a less predictable, unrelated event initiating new activity (i.e., marking a coarse event boundary). The results revealed an N400 effect of event boundary, with coarse boundaries eliciting larger N400 amplitudes than fine boundaries. In addition, there was an extended frontal positivity for elaborate descriptions involving multiple events compared to brief ones mentioning just one sub-event. This effect was largely driven by coarse event boundaries, that is, by target words referring to less predictable, unrelated events.

We interpret the two effects as indexing two stages of situation model construction: Retrieval of lexical semantic information (N400) and updating/revision of the situation model (P600) (see Brouwer et al., 2012).

While the functional interpretation of the N400 is still a matter of debate (e.g., Lau, Namyst, Fogel, & Delgado, 2016; Lau et al., 2008, for an overview see Kutas & Federmeier, 2011), there is growing consensus that N400 amplitude indexes processes associated with the ease of accessing and retrieving conceptual knowledge stored in long-term memory (e.g., Brouwer et al., 2012; Federmeier & Kutas, 1999; Kutas & Federmeier, 2000; Lau, Almeida, Hines, & Poeppel, 2009; Thornhill & van Petten, 2012). According to this view, N400 effects of predictability are generated by retrieval mechanisms and reflect the degree to which the preceding context activates conceptual knowledge associated with the eliciting word though mechanisms such as lexical or event schemas priming (e.g., Chow & Phillips, 2013; Chwilla & Kolk, 2005; Lau et al., 2016). In the current study, the N400 effect of event boundary suggests that the conceptual knowledge activated by the context primes fine boundary targets more than coarse boundary targets. Interestingly, the lack of an interaction with the type of event description suggests that the two types of description activate the same amount of knowledge (i.e., the same scripts or event schemas). It is this knowledge that facilitates retrieval of related (i.e., marking fine event boundaries) as opposed to unrelated (i.e., marking coarse event boundaries) events.

Rather than modulating the N400 amplitude, the length of the description influenced a late positive component, which has been associated with processes involved in discourse model updating (e.g. Brouwer et al., 2012; Burkhardt, 2006, 2007; Jouravlev et al., 2016; Schumacher, 2009, see also Donchin & Coles, 1988; Polich, 2007). More specifically, according to Brouwer et al., (2012), late positive components reflect the “construction, revision, or updating of a mental representation of what is being communicated” (Brouwer et al., 2012, p. 137; see also Brouwer, Crocker, Venhuizen, & Hoeks, 2016). Differences in amplitude, latencies, or scalp distributions reflect the different sub-processes that may underlie the construction of these mental representations (e.g., Brouwer & Hoeks, 2013). Frontal positivities have been found to be elicited in situations where comprehenders’ predictions about upcoming lexical items are disconfirmed (DeLong, Urbach, Groppe, & Kutas, 2011; Federmeier, Kutas, & Schul, 2010; Federmeier, Wlotko, Ochoa-Dewald, & Kutas, 2007; Thornhill & van Petten, 2012; Van Petten & Luka, 2012). In the present study, however, both brief and elaborate contexts were quite low-constraining. They did not generate high expectations for a specific word, possibly because they consisted of relatively brief (three-sentence) stories rather than rich and elaborate narratives.^4^ Furthermore, previous findings from research on predictive inferences in text comprehension (i.e., inferences about what should occur next in a story) have shown that such inferences are not typically constructed online, unless the predictable event is highly constrained by the context (e.g., Magliano, Baggett, Johnson, & Graesser, 1993; McKoon & Ratcliff, 1986; see also Graesser, Singer, & Trabasso, 1994). The frontal positivity in the current study is more likely related to expectations for a *type* of event boundary rather than for a specific event marking that boundary. Our cloze data show that unrelated events appeared more often following elaborate compared to brief contexts, suggesting that the activity described in elaborate contexts was more likely to be perceived as completed. Similar to what has been described for the perception of partial vs. complete events in Zacks (2010), in elaborate contexts script knowledge about the current activity becomes less helpful to generate accurate predictions about future events. It is in these situations that comprehenders anticipate they will need to initiate an updating process as soon as new information is encountered, as predicted by Event Segmentation Theory (Zacks et al., 2007). Consistent with this, we found that elaborate contexts elicited a (frontal) P600 effect compared to brief contexts, starting as early as 350 ms, that is, already in the N400 time window (see Fig. 2). We interpret this effect as indexing aspects of the updating process, consistent with previous work (see also Burmester, Spalek, & Watenburger, 2014; Kaan, Dallas, & Barkley, 2007; Wang & Schumacher, 2013). The fact that the effect becomes stronger for coarse event boundaries compared to fine event boundaries suggests that the updating process is more demanding when the target is fully unrelated to the activity described. This leads comprehenders to reset the old model and construct a new one (similar to the global updating processes outlined, for example, in Bailey & Zacks, 2015; Kurby & Zacks, 2012). For fine boundary targets, in contrast, the updating process is less demanding, either because the new model will contain more predictable information (recall that the cloze data showed a higher percentage of related compared to unrelated continuations in elaborate contexts, although unrelated events were produced more often than in brief contexts), or because comprehenders realize that the old model is still related to the new one and requires only incremental updating (see also, e.g., Zwaan, Langston, & Graesser, 1995; Zwaan et al., 1995).

Brief contexts, on the other hand, did not produce visible effects in the P600 component. This is consistent with the observation that brief contexts are more strongly predictive of events *continuing* the current activity, and are therefore less likely to trigger anticipation of (global) updating processes. Encountering an unexpected coarse boundary target in these contexts results in a more sustained N400 effect relative to fine boundary targets (see Fig. 1), reflecting enhanced difficulty in accessing and retrieving the unexpected event from long-term memory. This might have masked or, at the very least, delayed any updating process indexed by the P600. Thus, while EST makes no clear predictions for the mechanisms involved in this particular case, we suggest that our results are still broadly consistent with it.

In sum, the present findings provide electrophysiological support for EST (Zacks et al., 2007), which proposes that, for both event perception and narrative comprehension, mental representations of ‘what is happening now’ are updated in response to event boundaries (e.g., Speer & Zacks, 2005; Speer et al., 2007; Zacks et al., 2009).

The current results add to the growing body of evidence that processes associated with the construction and revision of situation models are reflected in the family of late positive components, as proposed by Brouwer et al., (2012). Future work is required to assess whether the (frontal) distribution of the effect can be taken to reflect one of the different sub-processes involved in the construction of these mental representations (see also Brouwer & Hoeks, 2013). On a more general level, the present study shows that comprehenders are sensitive to the structure of events, providing further evidence that stereotyped knowledge about everyday activities, so-called *event schemata* or *scripts* (Schank & Abelson, 1977), influences comprehenders’ expectations at early stages of processing.

### Electronic supplementary material

Below is the link to the electronic supplementary material.

(PDF 162 KB)

## Appendix

### Sample items with English translation

We report only elaborate contexts. Brief contexts included only the final action mentioned in elaborate contexts (see Table 1). Target words: fine boundary / coarse boundary.

1. Sebastian ist sehr hungrig. Er geht in die Küche, wo er sich erst Hühnchen, dann Sauce und dann Nudeln kocht. Dann beginnt er mit dem Verzehren / Wäschewaschen, wofür er 30 Minuten braucht. *(Sebastian is very hungry. He goes to the kitchen, where he cooks first chicken, then sauce, and then noodles. Then he begins to eat / do the laundry, for which he needs 30 minutes.)*
2. Christine ist mit der Vorlesung fertig. Sie geht in die Bibliothek, wo sie erst Magazine, dann Kataloge und dann Bücher einsortiert. Dann beginnt sie mit dem Lesen / Einkaufen, wofür sie … *(Christine has finished the lecture. She goes to the library, where she sorts first magazines, then catalogues, and then books. Then she begins to read / to shop, for which …)*
3. Katrin kommt von einer WG-Party. Sie geht in das Bad, wo sie erst ihren Nagellack, dann ihren Lidschatten und dann ihren Lippenstift entfernt. Dann beginnt sie mit dem Duschen / Bügeln, wofür sie … *(Katrin comes home from a WG-Party. She goes to the bathroom, where she removes first her nail polish, then her eye shadow, and then her lipstick. Then she starts to shower / to iron …)*
4. Brigittes Bettwäsche ist schmutzig. Sie geht in Schlafzimmer, wo sie erst die Bettdecke, dann das Kopfkissen und dann die Matratze neu bezieht. Dann beginnt sie mit dem Waschen / Blumengießen, … *(The bedclothes of Brigitte are dirty. She goes to the bedroom, where she re-covers first the blanket, then the pillow, and then the mattress. Then she starts to do the laundry / to water the flowers, …)*
5. Christian muss für eine Klausur lernen. Er setzt sich an den Schreibtisch, wo er sich erst ein Skript, dann ein Paper und dann ein Lehrbuch durchliest. Dann beginnt er mit dem Notieren / Kartenspielen, … *(Christian has to study for an exam. He sits down at his desk, where he reads first a script, then a paper, and then a textbook. Then he starts to take notes / to play cards, …)*
6. Mayte hat sich am Knie verletzt. Sie geht in die Apotheke, wo sie sich erst ein Desinfektionsmittel, dann eine Wundauflage und dann einen Verband kauft. Dann beginnt sie mit dem Verarzten / Eisessen, … Hat Mayte sich am Arm verletzt? *(Mayte has injured her knee. She goes to the pharmacy, where she buys first a germicide, then mull, and then a bandage. Then she starts to patch up / to eat an ice cream, … Has Mayte injured her arm?)*
7. Sabine bereitet das Weihnachtsessen vor. Sie geht in die Küche, wo sie erst das Rotkraut, dann die Kartoffeln und dann die Gans vorbereitet. Dann beginnt sie mit dem Füllen / Häkeln, … *(Sabine prepares the Christmas dinner. She goes to the kitchen, where she prepares first red cabbage, then potatoes, and then the goose. Then she starts to stuff / crochet, …)*
8. Hans möchte seine Frau überraschen. Er geht in die Küche, wo er erst das Besteck, dann die Gläser und dann die Teller spült. Dann beginnt er mit dem Wegräumen / Briefschreiben, … *(Hans wants to surprise his wife. He goes to the kitchen, where he cleans first the cutlery, then the glasses, and then the plates. Then he starts to clear / write a letter, …)*
9. Jan ist vom Laufen ganz verschwitzt. Er geht in die Dusche, wo er sich erst die Füße, dann die Arme und dann die Haare wäscht. Dann beginnt er mit dem Rasieren / Saugen, … *(Jan is all sweaty from running. He goes to the shower, where he washes first his feet, then his arms, and then his hair. Then he starts to shave / hoover, …)*
10. Sophia fährt in Urlaub. Sie geht zum Kleiderschrank, wo sie erst ihren Bikini, dann ihren Sommerrock und dann ihre Flip-Flops herausnimmt. Dann beginnt sie mit dem Packen / Fensterputzen, … *(Sophia goes on holidays. She goes to the wardrobe, where she takes first her bikini, then her skirt, and then her flip-flops. Then she starts to pack / clean the windows, …)*

## Footnotes

1 Sample size was determined on the basis of previous ERP literature on language; Luck (2014).

2 A linear mixed-effects model analysis of the data showed similar effects. We report ANOVAs in line with common practices in the ERP literature on language.

3 Note that the fact that the two elaborate conditions seem not to differ from each other in the P600 time window is likely due to component overlap. Since the processes underlying the N400 and the P600 may overlap in time, the amplitudes of the two components are likely to affect each other, as noticed in the literature (Brouwer & Crocker, 2017; Brouwer & Hoeks, 2013; Hagoort, 2003; Kutas, van Petten, & Kluender, 2006; Thornhill & van Petten, 2012, for a related proposal, see Tarren & Hell, 2014). In the 350-550 ms time window, coarse boundary targets are more negative than fine boundary targets (the N400 effect). This enhanced negativity may actually lead to a reduction of the positivity for coarse boundary targets in the 600–1000 ms time window. In other words, it is likely that, any P600 effect for coarse boundary targets compared to fine boundary targets is masked by the presence of an N400 effect.

4 Contextual constraint is defined as the cloze probability of the most highly preferred (or best) completion (e.g., Kutas & Hillyard, 1984), which in our items was only 35% following brief contexts and 32% following elaborate contexts.

## References

[CR1] Altmann, G. T. M., & Kamide, Y. (1999). Incremental interpretation at verbs: Restricting the domain of subsequent reference. *Cognition*, *73*(3), 247–264. 10.1016/s0010-0277(99)00059-1 10585516

[CR2] Bailey, H. R., & Zacks, J. M. (2015). Situation model updating in young and older adults: Global versus incremental mechanisms. *Psychology and Aging*, *30*(2), 232–2444. 10.1037/a0039081 PMC4451381 25938248

[CR3] Bestgen, Y., & Vonk, W. (2000). Temporal adverbial as segmentation markers in discourse comprehension. *Journal of Memory and Language*, *42*, 74–87.

[CR4] Bicknell, K., Elman, J. L., Hare, M., McRae, K., & Kutas, M. (2010). Effects of event knowledge in processing verbal arguments. *Journal of Memory and Language*, *63*, 489–505. 10.1016/j.jml.2010.08.004 PMC2976562 21076629

[CR5] Brouwer, H., & Crocker, M. (2017). On the proper treatment of the N400 and the P600 in language comprehension. *Frontiers in Psychology*, *8*, 1327. 10.3389/fpsyg.2017.01327 PMC5539129 28824506

[CR6] Brouwer, H., Crocker, M. W., Venhuizen, N. J., & Hoeks, J. C. J. (2016). A neurocomputational model of the N400 and the P600 in language processing. *Cognitive Science*, to appear. 10.1111/cogs.12461 PMC5484319 28000963

[CR7] Brouwer, H., Fitz, H., & Hoeks, J. (2012). Getting real about semantic illusions: Rethinking the functional role of the P600 in language comprehension. *Brain Research*, *1446*, 127–143. 10.1016/j.brainres.2012.01.055 22361114

[CR8] Brouwer, H., & Hoeks, J. C. J. (2013). A time and place for language comprehension: Mapping the N400 and the P600 to a minimal cortical network. *Frontiers in Human Neuroscience*, *7*, 758. 10.3389/fnhum.2013.00758 PMC3824103 24273505

[CR9] Burkhardt, P. (2006). Inferential bridging relations reveal distinct neural mechanisms: Evidence from event-related brain potentials. *Brain and Language*, *98*(2), 159–168. 10.1016/j.bandl.2006.04.005 16725188

[CR10] Burkhardt, P. (2007). The P600 reflects cost of new information in discourse memory. *NeuroReport*, *18*(17), 1851–1854. 10.1097/WNR.0b013e3282f1a999 18090325

[CR11] Burmester, J., Spalek, K., & Watenburger, I. (2014). Context updating during sentence comprehension: The effect of aboutness topic. *Brain and Language*, *137*, 62–76. 10.1016/j.bandl.2014.08.001 25156161

[CR12] Camblin, C. C., Gordon, P. C., & Swaab, T. Y. (2007). The interplay of discourse congruence and lexical association during sentence processing: Evidence from ERPs and eye tracking. *Journal of Memory and Language*, *56*, 103–128. 10.1016/j.jml.2006.07.005 PMC1766924 17218992

[CR13] Chow, W. Y., & Phillips, C. (2013). No semantic illusions in the “semantic P600” phenomenon: ERP evidence from Mandarin Chinese. *Brain Research*, *1506*, 76–93. 10.1016/j.brainres.2013.02.016 23422676

[CR14] Chwilla, D. J., & Kolk, H. H. J. (2005). Accessing world knowledge: Evidence from N400 and reaction time priming. *Cognitive Brain Research*, *25*, 589–606. 10.1016/j.cogbrainres.2005.08.011 16202570

[CR15] Dambacher, M., Kliegl, R., Hofmann, M., & Jacobs, A. M. (2006). Frequency and predictability effects on event-related potentials during reading. *Brain Research*, *1084*(1), 89–103. 10.1016/j.brainres.2006.02.010 16545344

[CR16] DeLong, K. A., Urbach, T. P., Groppe, D. M., & Kutas, M. (2011). Overlapping dual ERP responses to low cloze probability sentence continuations. *Psychophysiology*, *48*(9), 1203–1027. 10.1111/j.1469-8986.2011.01199.x 10.1111/j.1469-8986.2011.01199.x PMC3131420 21457275

[CR17] Donchin, E., & Coles, M. G. H. (1988). Is the P300 component a manifestation of cognitive updating? *Behavioral and Brain Sciences*, *11*(3), 357–427.

[CR18] Federmeier, K. D., & Kutas, M. (1999). A rose by any other name: Long-term memory structure and sentence processing. *Journal of Memory and Language*, *41*, 469–495.

[CR19] Federmeier, K. D., Kutas, M., & Schul, R. (2010). Age-related and individual differences in the use of prediction during language comprehension. *Brain and Language*, *115*(3), 149–161. 10.1016/j.bandl.2010.07.006 PMC2975864 20728207

[CR20] Federmeier, K. D., Wlotko, E. W., Ochoa-Dewald, E. D., & Kutas, M. (2007). Multiple effects of sentential constraint on word processing. *Brain Research*, *1146*, 75–84. 10.1016/j.brainres.2006.06.101 PMC2704150 16901469

[CR21] George, M. S. S., Mannes, S., & Hoffman, J. E. (1997). Individual differences in inference generation: An ERP analysis. *Journal of Cognitive Neuroscience*, *9*(6), 776–787. 10.1162/jocn.1997.9.6.776 23964599

[CR22] Gernsbacher, M. A. (1990). *Language comprehension as structure building*. Hillsdale: Erlbaum.

[CR23] Graesser, A. C., Singer, M., & Trabasso, T. (1994). Constructing inferences during narrative text comprehension. *Psychological Review*, *101*(3), 371–395. 10.1037/0033-295x.101.3.371 7938337

[CR24] Hagoort, P. (2003). Interplay between syntax and semantics during sentence comprehension: ERP effects of combining syntactic and semantic violations. *Journal of Cognitive Neuroscience*, *15*(6), 883–899. 10.1162/089892903322370807 14511541

[CR25] Hagoort, P., Brown, C., & Groothusen, J. (1993). The syntactic positive shift (SPS) as an ERP measure of syntactic processing. *Language and Cognitive Processes*, *8*, 439–483.

[CR26] Hare, M., Jones, M., Thomson, C., Kelly, S., & McRae, K. (2009). Activating event knowledge. *Cognition*, *111*, 151–167. 10.1016/j.cognition.2009.01.009 PMC2831639 19298961

[CR27] Johnson-Laird, P.N. (1983). *Mental models: Towards a cognitive science of language, inference, and consciousness*. Harvard: Harvard University Press.

[CR28] Jouravlev, O., Stearns, L., Bergen, L., Eddy, M., Gibson, E., & Fedorenko, E. (2016). Processing temporal presupposition: An event-related potential study. *Language, Cognition and Neuroscience*, *31*(10), 1245–1256.

[CR29] Kaan, E., Dallas, A. C., & Barkley, C. M. (2007). Processing bare quantifiers in discourse. *Brain Research*, *1146*, 199–209. 10.1016/j.brainres.2006.09.060 PMC1900382 17070788

[CR30] Kintsch, W., & van Dijk, T. A. (1978). Toward a model of text comprehension and production. *Psychological Review*, *85*, 363–394.

[CR31] Kurby, C. A., & Zacks, J. M. (2012). Starting from scratch and building brick by brick in comprehension. *Memory and Cognition*, *40*(5), 812–826. 10.3758/s13421-011-0179-8 PMC4190164 22282158

[CR32] Kutas, M., & Federmeier, K. D. (2000). Electrophysiology reveals semantic memory use in language comprehension. *Trends in Cognitive Sciences*, *4*(12), 463–470. 10.1016/s1364-6613(00)01560-6 11115760

[CR33] Kutas, M., & Federmeier, K. D. (2011). Thirty years and counting: Finding meaning in the N400 component of the event-related brain potential (ERP). *Annual Review of Psychology*, *62*, 621–647. 10.1146/annurev.psych.093008.131123 PMC4052444 20809790

[CR34] Kutas, M., & Hillyard, S. A. (1984). Brain potentials during reading reflect word expectancy and semantic association. *Nature*, *307*, 161–163. 10.1038/307161a0 6690995

[CR35] Kutas, M., van Petten, C., & Kluender, R. (2006). Psycholinguistics electrified II: 1994–2005. In M. J. Traxler, & M. A. Gernsbacher (Eds.) *Handbook of psycholinguistics: 2nd edition* (pp. 659–724). New York: Elsevier.

[CR36] Lau, E. F., Almeida, D., Hines, P. C., & Poeppel, D. (2009). A lexical basis for N400 context effects: Evidence from MEG. *Brain and Language*, *111*, 161–172. 10.1016/j.bandl.2009.08.007 PMC2783912 19815267

[CR37] Lau, E. F., Namyst, A., Fogel, A., & Delgado, T. (2016). A direct comparison of N400 effects of predictability and incongruity in adjective-noun combination. *Collabra*, *2*(1), 1–19.

[CR38] Lau, E. F., Phillips, C., & Poeppel, D. (2008). A cortical network for semantics: (De)constructing the N400. *Nature Reviews Neuvoscience*, *9*, 920–933. 10.1038/nrn2532 19020511

[CR39] Luck, S. J. (2014). *An introduction to the event-related potential technique (2nd ed.)* Cambridge, MA: The MIT Press.

[CR40] Magliano, J. P., Baggett, W. B., Johnson, B. K., & Graesser, A. C. (1993). The time course of generating causal antecedent and causal consequence inferences. *Discourse Processes*, *16*, 35–53.

[CR41] Magliano, J. P., Miller, J., & Zwaan, R. A. (2001). Indexing space and time in film understanding. *Applied Cognitive Psychology*, *15*, 533–545.

[CR42] Matsuki, K., Chow, T., Hare, M., Elman, H. L., Scheepers, C., & McRae, K. (2011). Event-based plausibility immediately influences on-line language comprehension. Journal of Experimental Psychology: Learning. *Memory, and Cognition*, *37*(4), 913–934. 10.1037/a0022964 PMC3130834 21517222

[CR43] McKoon, G., & Ratcliff, R. (1986). Inferences about predictable events. Journal of Experimental Psychology: Learning. *Memory, and Cognition*, *12*(1), 82–91. 10.1037//0278-7393.12.1.82 2949049

[CR44] McRae, K., Hare, M., Elman, J. L., & Ferretti, T. R. (2005). A basis for generating expectancies for verbs from nouns. *Memory and Cognition*, *33*, 1174–1184. 10.3758/bf03193221 16532852

[CR45] Metusalem, R., Kutas, M., Hare, M., McRae, K., & Elman, J. L. (2012). Generalized event knowledge activation during online sentence comprehension. *Journal of Memory and Language*, *66*, 545–567. 10.1016/j.jml.2012.01.001 PMC3375826 22711976

[CR46] Otten, M., & van Berkum, J. J. A. (2007). What makes a discourse constraining? Comparing the effects of discourse message and scenario fit on the discourse-dependent N400 effect. *Brain Research*, *1153*, 166–177. 10.1016/j.brainres.2007.03.058 17466281

[CR47] Polich, J. (2007). Updating P300: An integrative theory of P3a and P3b. *Clinical Neuropsysiology: Official Journal of the International Federation of Clinical Neurophysiology*, *118*(10), 2128–2148. 10.1016/j.clinph.2007.04.019 PMC2715154 17573239

[CR48] Rinck, M., & Weber, U. (2003). Who when where: An experimental test of the event-indexing model. *Memory and Cognition*, *31*, 1284–292. 10.3758/bf03195811 15058689

[CR49] Rugg, M. D. (1990). Event-related brain potentials dissociate repetition effects on high and low-frequency words. *Memory and Cognition*, *18*(4), 367–379. 10.3758/bf03197126 2381316

[CR50] Schank, R. C., & Abelson, R. P. (1977). *Scripts, plans, goals and understanding: An inquire into human knowledge structures*. Hillsdale: L. Erlbaum.

[CR51] Schumacher, P. B. (2009). Definiteness marking shows late effects during discourse processing: Evidence from ERPs. In S. L. Devi, A. Branco, & R. Mitkov (Eds.), (Vol. 5847, pp. 91–106). Berlin: Springer.

[CR52] Schumacher, P. B., & Hung, Y. C. (2012). Positional influences on information packaging: Insights from topological fields in German. *Journal of Memory and Language*, *67*, 295–310.

[CR53] Sitnikova, T., Holcomb, P. J., Kiyonaga, K. A., & Kuperberg, G. R. (2008). Two neurocognitive mechanisms of semantic integration during comprehension of visual real-world events. *Journal of Cognitive Neuroscience*, *20*(11), 2037–2057. 10.1162/jocn.2008.20143 PMC2673092 18416681

[CR54] Speer, N. K., Reynolds, J. R., Swallow, K. M., & Zacks, J. M. (2009). Reading stories activates neural representations of perceptual and motor experiences. *Psychological Science*, *20*, 989–999. 10.1111/j.1467-9280.2009.02397.x PMC2819196 19572969

[CR55] Speer, N. K., & Zacks, J. M. (2005). Temporal changes as event boundaries: Processing and memory consequences of narrative time shifts. *Journal of Memory and Language*, *53*, 125–140.

[CR56] Speer, N. K., Zacks, J. M., & Reynolds, J. R. (2007). Human brain activity time-locked to narrative event boundaries. *Psychological Science*, *18*, 449–455. 10.1111/j.1467-9280.2007.01920.x 17576286

[CR57] Tarren, D., & Hell, J. G. V. (2014). ERPs reveal individual differences in morphosyntactic processing. *Neurpshycologia*, *56*, 289–301. 10.1016/j.neuropsychologia.2014.02.002 24530237

[CR58] Thornhill, F. E., & van Petten, C. (2012). Lexical versus conceptual anticipation during sentence processing: Frontal and N400 components. *International Journal of Psychophysiology*, *83*, 382–392. 10.1016/j.ijpsycho.2011.12.007 22226800

[CR59] van Berkum, J. J. A., Hagoort, P., & Brown, M. (1999). Semantic integration in sentences and discourse: Evidence from the N400. *Journal of Cognitive Neuroscience*, *11*(6), 657–671. 10.1162/089892999563724 10601747

[CR60] Van Dijk, T. A., & Kintsch, W. (1983). *Strategies of discourse comprehension*. New York: Academic Press.

[CR61] Van Petten, C., & Kutas, M. (1990). Interaction between sentence context and word frequency in event-related brain potential. *Memory and Cognition*, *18*(4), 380–393. 10.3758/bf03197127 2381317

[CR62] Van Petten, C., & Luka, B. (2012). Prediction during language comprehension: Benefits, costs, and ERP components. *International Journal of Psychophysiology*, *83*(1), 176–190. 10.1016/j.ijpsycho.2011.09.015 22019481

[CR63] Wang, L., & Schumacher, P. B. (2013). New is not always costly: Evidence from online processing of topic and contrast in Japanese. *Frontiers in Psychology*, *4*, 363. 10.3389/fpsyg.2013.00363 PMC3695390 23825466

[CR64] Whitney, C., Huber, W., Klann, J., Weis, S., Krach, S., & Kircher, T. (2009). Neural correlates of narrative shifts during auditory story comprehension. *NeuroImage*, *47*(1), 360–366. 10.1016/j.neuroimage.2009.04.037 19376237

[CR65] Zacks, J. M. (2010). How we organize our experience into events. *Psychological Science Agenda*, *24*(4).

[CR66] Zacks, J. M., Braver, T. S., Sheridan, M. A., Donaldson, D. I., Snyder, A. Z., Ollinger, J.M., ..., & Raichie, M. E. (2001). Human brain activity time-locked to perceptual event boundaries. *Nature Neuroscience*, *4*, 651–655. 10.1038/88486 11369948

[CR67] Zacks, J. M., Speer, N. K., & Reynolds, J. R. (2009). Segmentation in reading and film comprehension. *Journal of Experimental Psychology: General*, *138*, 307–327. 10.1037/a0015305 PMC8710938 19397386

[CR68] Zacks, J. M., Speer, N. K., Swallow, K. M., Braver, T. S., & Reynolds, J. R. (2007). Event perception: A mind/brain perspective. *Psychological Bulletin*, *133*, 273–293. 10.1037/0033-2909.133.2.273 PMC2852534 17338600

[CR69] Zacks, J. M., & Swallow, K. M. (2007). Event segmentation. *Current Directions in Psychological Science*, *16*, 80–84. 10.1111/j.1467-8721.2007.00480.x PMC3314399 22468032

[CR70] Zwaan, R. A. (1996). Processing narrative time shifts. *Journal of Experimental Psychology: Learning, Memory, and Cognition*, *22*(5), 1196–1207.

[CR71] Zwaan, R. A., Langston, M., & Graesser, A. C. (1995). The construction of situation models in narrative comprehension: An event-indexing model. *Psychological Science*, *6*, 292–297.

[CR72] Zwaan, R. A., Magliano, J. P., & Graesser, A. C. (1995). Dimensions of situation model construction in narrative comprehension. Journal of Experimental Psychology: Learning. *Memory, and Cognition*, *21*, 386–397.

[CR73] Zwaan, R. A., & Radvansky, G. A. (1998). Situation models in language comprehension and memory. *Psychonomic Bulletin*, *123*(2), 162–185. 10.1037/0033-2909.123.2.162 9522683

