# Supplementary material for: On the predictability of event boundaries in discourse: An ERP investigation
Source: Mem Cognit. 2017 Nov 20;46(2):315–25. doi: 10.3758/s13421-017-0766-4 (PMC5809541; doi:10.3758/s13421-017-0766-4)
Supplement: Supplementary file 1 — (PDF 162 KB) [file 13421_2017_766_MOESM1_ESM.pdf]

Full list of experimental items

(a. Brief description; b. Elaborate description; T = Target sentence; Target word underlined: <u>Fine boundary</u> / <u>Coarse boundary; Q = Question)</u>

1. Sebastian ist sehr hungrig.
a. Er geht in die Küche, wo er sich Nudeln kocht.
b. Er geht in die Küche, wo er sich erst Hühnchen, dann Sauce und dann Nudeln kocht.
T: Dann beginnt er mit dem <u>Verzehren</u>/ <u>Wäschewaschen</u>, wofür er 30 Minuten braucht.

2. Christine ist mit der Vorlesung fertig.
a. Sie geht in die Bibliothek, wo sie Bücher einsortiert.
b. Sie geht in die Bibliothek, wo sie erst Magazine, dann Kataloge und dann Bücher einsortiert.
T: Dann beginnt sie mit dem <u>Lesen</u> / <u>Einkaufen</u>, wofür sie 30 Minuten braucht.

3. Jörn ist mit dem Frühstück fertig.
a. Er geht in die Küche, wo er Teller abwäscht.
b. Er geht in die Küche, wo er erst Tassen, dann Besteck und dann Teller abwäscht.
T: Dann beginnt er mit dem <u>Abtrocknen</u> / <u>Joggen</u>, wofür er 30 Minuten braucht.

4. Luca will sich die Zeit vertreiben.
a. Er geht in sein Schlafzimmer, wo er den Computer anmacht.
b. Er geht in sein Schlafzimmer, wo er erst den Router, dann die Lautsprecher und dann den Computer anmacht.
T: Dann beginnt er mit dem <u>Spielen</u> / <u>Bügeln</u>, wofür er 30 Minuten braucht.
Q: Schaltet Luca den Computer ein?

5. Helena geht auf ein Festival.
a. Sie geht zu den Ständen, wo sie sich Bier holt.
b. Sie geht zu den Ständen, wo sie sich erst Streichhölzer, dann Zigaretten und dann Bier holt.
T: Dann beginnt sie mit dem <u>Tanzen</u> / <u>Zeltabbauen</u>, wofür sie 30 Minuten braucht.

6. Ilka füttert die Hühner.
a. Sie geht in den Stall, wo sie Maiskörner verstreut.
b. Sie geht in den Stall, wo sie erst Weizen, dann Heu und dann Maiskörner verstreut.

T: Dann beginnt sie mit dem Ausmisten / Schlachten, wofür sie 30 Minuten braucht.

7. Katrin kommt von einer WG-Party.
a. Sie geht in das Bad, wo sie ihren Lippenstift entfernt.
b. Sie geht in das Bad, wo sie erst ihren Nagellack, dann ihren Lidschatten und dann ihren Lippenstift entfernt.
T: Dann beginnt sie mit dem Duschen / Bügeln, wofür sie 30 Minuten braucht.

8. Frederik geht schick essen.
a. Er geht in das Badezimmer, wo er seine Frisur überprüft.
b. Er geht in das Badezimmer, wo er erst seinen Kragen, dann seine Krawatte und dann seine Frisur überprüft.
T: Dann beginnt er mit dem Kämmen / Saugen, wofür er 5 Minuten braucht.
Q: Geht Frederik in die Imbissbude?

9. Nadja versucht den Schulstoff aufzuholen.
a. Sie geht an ihren Schreibtisch, wo sie ihr Heft herausholt.
b. Sie geht an ihren Schreibtisch, wo sie erst ihren Stift, dann ihren Radiergummi und dann ihr Heft herausholt.
T: Dann beginnt sie mit dem Lernen / Bügeln, wofür sie 30 Minuten braucht.

10. Brigittes Bettwäsche ist schmutzig.
a. Sie geht ins Schlafzimmer, wo sie die Matratze neu bezieht.
b. Sie geht in Schlafzimmer, wo sie erst die Bettdecke, dann das Kopfkissen und dann die Matratze neu bezieht.
T: Dann beginnt sie mit dem Waschen / Blumengießen, wofür sie 30 Minuten braucht.

11. Christian muss für eine Klausur lernen.
a. Er setzt sich an den Schreibtisch, wo er sich ein Lehrbuch durchliest.
b. Er setzt sich an den Schreibtisch, wo er sich erst ein Skript, dann ein Paper und dann ein Lehrbuch durchliest.
T: Dann beginnt er mit dem Notieren / Kartenspielen, wofür er 30 Minuten braucht.

12. Isabell ist fußballbegeistert.
a. Sie geht auf den Fußballplatz, wo sie den Torschuss trainiert.
b. Sie geht auf den Fußballplatz, wo sie erst die Ballannahme, dann das Passspiel und dann den Torschuss trainiert.
T: Dann beginnt sie mit dem Dribbeln / Shoppen, wofür sie 30 Minuten braucht.
Q: Mag Isabell Fußball?

13. Lara ist gut in Handarbeiten.
a. Sie setzt sich aufs Sofa, wo sie eine Jacke näht.
b. Sie setzt sich aufs Sofa, wo sie erst eine Tasche, dann eine Hose und dann eine Jacke näht.
T: Dann beginnt sie mit dem Stricken / Kaffeekochen, wofür sie 30 Minuten braucht.

14. Jessica freut sich auf Weihnachten.
a. Sie geht ins Wohnzimmer, wo sie eine Lichterkette aufhängt.
b. Sie geht ins Wohnzimmer, wo sie erst einen Strohstern, dann Lametta und dann eine Lichterkette aufhängt.
T: Dann beginnt sie mit dem Dekorieren / Kaffeetrinken, wofür sie 30 Minuten braucht.

15. Anina ist eine Leseratte.
a. Sie setzt sich in den Lesesessel, wo sie einen Roman liest.
b. Sie setzt sich in den Lesesessel, wo sie erst ein Sachbuch, dann einen Krimi und dann einen Roman liest.
T: Dann beginnt sie mit dem Nachdenken / Kuchenbacken, wofür sie 30 Minuten braucht.

16. Mayte hat sich am Knie verletzt.
a. Sie geht in die Apotheke, wo sie sich einen Verband kauft.
b. Sie geht in die Apotheke, wo sie sich erst ein Desinfektionsmittel, dann eine Wundauflage und dann einen Verband kauft.
T: Dann beginnt sie mit dem Verarzten / Eisessen, wofür sie 10 Minuten braucht.
Q: Hat Mayte sich am Arm verletzt?

17. Jörg macht Ferien auf dem Bauernhof.
a. Er geht auf die Weide, wo er ein Pony striegelt.
b. Er geht auf die Weide, wo er erst einen Hengst, dann eine Stute und dann ein Pony striegelt.
T: Dann beginnt er mit dem Reiten / Angeln, wofür er 30 Minuten braucht.

18. Franz vermisst seine Freundin.
a. Er setzt sich aufs Bett, wo er ihr einen Brief schreibt.
b. Er setzt sich aufs Bett, wo er ihr erst eine SMS, dann eine Mail und dann einen Brief schreibt.
T: Dann beginnt er mit dem Träumen / Videospielen, wofür er 1 Stunde braucht.

19. Samira will zu Weihnachten Plätzchen verschenken.
a. Sie geht in die Küche, wo sie Lebkuchen backt.
b. Sie geht in die Küche, wo sie erst Makronen, dann Spritzgebäck und dann Lebkuchen backt.
T: Dann beginnt sie mit dem Eintüten / Zeitunglesen, wofür sie 30 Minuten braucht.

20. Silke hat nichts mehr zum Anziehen übrig.
a. Sie geht in die Waschküche, wo sie die dunkle Wäsche wäscht.
b. Sie geht in die Waschküche, wo sie erst die Feinwäsche, dann die helle und dann die dunkle Wäsche wäscht.
T: Dann beginnt sie mit dem Aufhängen / Joggen, wofür sie 30 Minuten braucht.
Q: Wäscht Silke ihre Wäsche im Badezimmer?

21. Torben will wandern.
a. Er geht ins Wohnzimmer, wo er seine Karte bereitlegt.
b. Er geht ins Wohnzimmer, wo er erst seinen Rucksack, dann seinen Wanderstock und dann seine Karte bereitlegt.
T: Dann beginnt er mit dem Planen / Wäschewaschen, wofür er 30 Minuten braucht.

22. Rebecca geht shoppen.
a. Sie geht in das Bekleidungsgeschäft, wo sie Schuhe anprobiert.
b. Sie geht in das Bekleidungsgeschäft, wo sie erst Socken, dann Hosen und dann Schuhe anprobiert.
T: Dann beginnt sie mit dem Aussuchen / Nachhausegehen, wofür sie 30 Minuten braucht.

23. Rolf treibt gerne Sport.
a. Er geht ins Fitnessstudio, wo er seine Adduktoren dehnt.
b. Er geht ins Fitnessstudio, wo er erst seine Arme, dann seine Beine und dann seine Adduktoren dehnt.
T: Dann beginnt er mit dem Trainieren / Duschen, wofür er 30 Minuten braucht.

24. Max macht sich bettfertig.
a. Er geht ins Badezimmer, wo er sich das Gesicht wäscht.
b. Er geht ins Badezimmer, wo er sich erst die Hände, dann den Hals und dann das Gesicht wäscht.
T: Dann beginnt er mit dem Zähneputzen / Entkleiden, wofür er 5 Minuten braucht.
Q: Macht sich Max bettfertig?

25. Marco veranstaltet einen Spieleabend.

a. Er geht ins Wohnzimmer, wo er die Spiele auf den Tisch stellt.
b. Er geht ins Wohnzimmer, wo er erst das Knabberzeug, dann die Getränke und dann die Spiele auf den Tisch stellt.
T: Dann beginnt er mit dem Warten / Zigarettenkaufen, wofür er 15 Minuten braucht.

26. Viktorias Auto ist dreckig.
a. Sie fährt zur Waschstraße, wo sie den Kofferraum saugt.
b. Sie fährt zur Waschstraße, wo sie erst den Fußraum, dann die Fußmatten und dann den Kofferraum saugt.
T: Dann beginnt sie mit dem Putzen / Brillenputzen, wofür sie 30 Minuten braucht.

27. Mario will eine längere Strecke mit dem Auto fahren.
a. Er fährt zur Tankstelle, wo er die Tankanzeige überprüft.
b. Er fährt zur Tankstelle, wo er erst den Reifendruck, dann die Autobatterie und dann die Tankanzeige überprüft.
T: Dann beginnt er mit dem Zapfen / Einchecken, wofür er 2 Minuten braucht.

28. Elisa geht Weihnachtsgeschenke kaufen.
a. Sie geht ins Einkaufszentrum, wo sie ein Geschenk für ihre Schwester kauft.
b. Sie geht ins Einkaufszentrum, wo sie erst ein Geschenk für ihren Vater, dann ihre Mutter und dann ihre Schwester kauft.
T: Dann beginnt sie mit dem Einpacken / Baumschmücken, wofür sie 30 Minuten braucht.
Q: Kauft Elisa ein Geschenk für ihren Bruder?

28. Elisa geht Weihnachtsgeschenke kaufen.
a. Sie geht ins Einkaufszentrum, wo sie ein Geschenk für ihre Schwester kauft.
b. Sie geht ins Einkaufszentrum, wo sie erst ein Geschenk für ihren Vater, dann ihre Mutter und dann ihre Schwester kauft.
T: Dann beginnt sie mit dem Baumschmücken / Fernsehen, wofür sie 30 Minuten braucht.

29. Sven hat Langeweile.
a. Er geht ins Wohnzimmer, wo er auf dem Sofa herumsitzt.
b. Er geht ins Wohnzimmer, wo er erst auf dem Stuhl, dann auf dem Sessel und dann auf dem Sofa herumsitzt.
T: Dann beginnt er mit dem Fernsehen / Abwaschen, wofür er 30 Minuten braucht.

30. Steffen bastelt gerne.

a. Er geht ins Wohnzimmer, wo er sich Pappe nimmt.
b. Er geht ins Wohnzimmer, wo er sich erst eine Schere, dann ein Lineal und dann Pappe nimmt.

T: Dann beginnt er mit dem Ausschneiden / Abendessen, wofür er 5 Minuten braucht.

31. Sabine bereitet das Weihnachtsessen vor.

a. Sie geht in die Küche, wo sie die Gans vorbereitet.
b. Sie geht in die Küche, wo sie erst das Rotkraut, dann die Kartoffeln und dann die Gans vorbereitet.

T: Dann beginnt sie mit dem Füllen / Häkeln, wofür sie 10 Minuten braucht.

32. Frank möchte an einem Tanzkurs teilnehmen.

a. Er fährt zum Tanzstudio, wo er sich Schuhe ausleiht.
b. Er fährt zum Tanzstudio, wo er sich erst eine Sporthose, dann ein Schweißband und dann Schuhe ausleiht.

T: Dann beginnt er mit dem Anziehen / Wassertrinken, wofür er 5 Minuten braucht.
Q: Leiht Frank sich Schuhe aus?

33. Carsten möchte ein Videospiel spielen.

a. Er geht ins Wohnzimmer, wo er den Fernseher einschaltet.
b. Er geht ins Wohnzimmer, wo er erst die Konsole, dann die Stereoanlage und dann den Fernseher einschaltet.

T: Dann beginnt er zu Zocken / Schlummern, wofür er 30 Minuten braucht.

34. Hans möchte seine Frau überraschen.

a. Er geht in die Küche, wo er die Teller spült.
b. Er geht in die Küche, wo er erst das Besteck, dann die Gläser und dann die Teller spült.

T: Dann beginnt er mit dem Wegräumen / Briefschreiben, wofür er 10 Minuten braucht.

35. Marie muss für eine Englischarbeit lernen.

a. Sie geht in ihr Zimmer, wo sie die Vokabeln durchgeht.
b. Sie geht in ihr Zimmer, wo sie erst die Vergangenheitsformen, dann die Grammatik und dann die Vokabeln durchgeht.

T: Dann beginnt sie mit dem Wiederholen / Kaffeekochen, wofür sie 30 Minuten braucht.

36. Timo will neue Leute kennenlernen.

a. Er geht in eine Bar, wo er eine junge Frau anspricht.
b. Er geht in eine Bar, wo er erst ein Pärchen, dann einen Mann und dann eine junge Frau anspricht.

T: Dann beginnt er mit dem <u>Flirten</u> / <u>Zeitungslesen</u>, wofür er 30 Minuten braucht.
Q: Spricht Timo eine junge Frau an?

37. Magda macht den Frühjahrsputz.
a. Sie geht ins Wohnzimmer, wo sie die Gläser abstaubt.
b. Sie geht ins Wohnzimmer, wo sie erst den Tisch, dann das Regal und dann die Gläser abstaubt.

T: Dann beginnt sie mit dem <u>Saugen</u> / <u>Essenmachen</u>, wofür sie 30 Minuten braucht.

38. Irene will mit ihren Freundinnen ausgehen.
a. Sie geht ins Badezimmer, wo sie sich die Haare wäscht.
b. Sie geht ins Badezimmer, wo sie sich erst das Gesicht, dann die Füße und dann die Haare wäscht.

T: Dann beginnt sie mit dem <u>Föhnen</u> / <u>Treppensteigen</u>, wofür sie 10 Minuten braucht.

39. Torsten möchte Ordnung schaffen.
a. Er geht in sein Zimmer, wo er den Aschenbecher ausleert.
b. Er geht in sein Zimmer, wo er erst den Papierkorb, dann die Getränkereste und dann den Aschenbecher ausleert.

T: Dann beginnt er mit dem <u>Staubsaugen</u> / <u>Türreparieren</u>, wofür er 30 Minuten braucht.

40. Danielo möchte seinem Lehrer schreiben.
a. Er geht in sein Zimmer, wo er den Brieftext verfasst.
b. Er geht in sein Zimmer, wo er erst den Briefkopf, dann den Gruß und dann den Brieftext verfasst.

T: Dann beginnt er mit dem <u>Abschicken</u> / <u>Ausmisten</u>, wofür er 30 Minuten braucht.
Q: Schreibt Danielo seiner Großmutter?

41. Erika möchte ihre Mutter anrufen.
a. Sie geht in ihr Arbeitszimmer, wo sie das Telefon sucht.
b. Sie geht in ihr Arbeitszimmer, wo sie erst das Adressbuch, dann einen Stift und dann das Telefon sucht.

T: Dann beginnt sie mit dem <u>Wählen</u> / <u>Werkeln</u>, wofür sie 2 Minuten braucht.

42. Marc ist Schreiner.

a. Er geht in die Werkstatt, wo er sich Bretter nimmt.
b. Er geht in die Werkstatt, wo er sich erst Schrauben, dann Muttern und dann Bretter nimmt.
T: Dann beginnt er mit dem <u>Sägen</u> / <u>Müllwegbringen</u>, wofür er 30 Minuten braucht.

43. Sylvia ist Kindergärtnerin.
a. Sie geht ins Zimmer, wo sie die Bauklötze zusammensucht.
b. Sie geht ins Zimmer, wo sie erst die Knete, dann die Puppen und dann die Bauklötze zusammensucht.
T: Dann beginnt sie mit dem <u>Spielen</u> / <u>Rauchen</u>, wofür sie 20 Minuten braucht.

44. Karina ist Frisörin.
a. Sie geht in den Salon, wo sie einer Frau die Haare färbt.
b. Sie geht in den Salon, wo sie erst einem Teenager, dann einem Mann und dann einer Frau die Haare färbt.
T: Dann beginnt sie mit dem <u>Schneiden</u> / <u>Einkaufen</u>, wofür sie 5 Minuten braucht.
Q: Färbt Karina einer Frau die Haare?

45. Olaf ist Fußballtrainer.
a. Er geht in den Umkleideraum, wo er die Spielstrategie erklärt.
b. Er geht in den Umkleideraum, wo er erst die Schwächen, dann Stärken und dann die Spielstrategie erklärt.
T: Dann beginnt er mit dem <u>Motivieren</u> / <u>Würstchenessen</u>, wofür er 30 Minuten braucht.

46. Helga möchte ihre Kleidung waschen.
a. Sie geht in das Badezimmer, wo sie die Maschine mit der Wäsche befüllt.
b. Sie geht in das Badezimmer, wo sie die Maschine erst mit dem Weichspüler, dann dem Waschmittel und dann der Wäsche befüllt.
T: Dann beginnt sie mit dem <u>Einschalten</u> / <u>Staubsaugen</u>, wofür sie 1 Minuten braucht.

47. Olga möchte ein Buch lesen.
a. Sie geht ins Wohnzimmer, wo sie ihr Lieblingsbuch holt.
b. Sie geht ins Wohnzimmer, wo sie erst ein Glas Wein, dann Schokolade und dann ihr Lieblingsbuch holt.
T: Dann beginnt sie mit dem <u>Schmökern</u> / <u>Singen</u>, wofür sie 30 Minuten braucht.

48. Sandro möchte ein Fußballspiel sehen.
a. Er geht ins Wohnzimmer, wo er die Fernbedienung holt.

b. Er geht ins Wohnzimmer, wo er erst die Erdnussflips, dann das Bier und dann die Fernbedienung holt.
T: Dann beginnt er mit dem Fernsehen / Baden, wofür er 30 Minuten braucht.
Q: Braucht Sandro dafür mehr als zwanzig Minuten?

49. Anne möchte ins Ausland fliegen.
a. Sie fährt zum Flughafen, wo sie in das Flugzeug einsteigt.
b. Sie fährt zum Flughafen, wo sie erst in den Bus, dann in das Shuttle und dann in das Flugzeug einsteigt.
T: Dann beginnt sie mit dem Platzsuchen / Verzollen, wofür sie 5 Minuten braucht.

50. Linus soll seine Hausaufgaben fertigstellen.
a. Er geht in sein Arbeitszimmer, wo er eine Hausarbeit fertig schreibt.
b. Er geht in sein Arbeitszimmer, wo er erst einen Aufsatz, dann ein Gedicht und dann eine Hausarbeit fertig schreibt.
T: Dann beginnt er mit dem Korrigieren / Getränkeholen, wofür er 30 Minuten braucht.

51. Jan ist vom Laufen ganz verschwitzt.
a. Er geht in die Dusche, wo er sich die Haare wäscht.
b. Er geht in die Dusche, wo er sich erst die Füße, dann die Arme und dann die Haare wäscht.
T: Dann beginnt er mit dem Rasieren / Saugen, wofür er 10 Minuten braucht.

52. Franziska will sich fit halten.
a. Sie geht in die Sporthalle, wo sie die Arme trainiert.
b. Sie geht in die Sporthalle, wo sie erst den Bauch, dann die Arme und dann die Beine trainiert.
T: Dann beginnt sie mit dem Joggen / Kochen, wofür sie 30 Minuten braucht.
Q: Trainiert Franziska im Fitnessstudio?

53. Daniel pflegt gerne seine Pflanzen.
a. Er geht in den Garten, wo er die Bäume wässert.
b. Er geht in den Garten, wo er erst die Büsche, dann die Blumen und dann die Bäume wässert.
T: Dann beginnt er mit dem Rasenmähen / Telefonieren, wofür er 30 Minuten braucht.

54. Anja hat Heißhunger auf etwas Süßes.
a. Sie geht an den Kühlschrank, wo sie den Kuchen herausholt.

b. Sie geht an den Kühlschrank, wo sie erst die Schokolade, dann den Pudding und dann den Kuchen herausholt.
T: Dann beginnt sie mit dem Falten / Rasieren wofür sie 30 Minuten braucht.

55. Julia hat die Wäsche abgehängt.
a. Sie geht ins Schlafzimmer, wo sie die Hemden bügelt.
b. Sie geht ins Schlafzimmer, wo sie erst die Socken, dann die Hosen und dann die Hemden bügelt.
T: Dann beginnt sie mit dem Aufräumen / Geigespielen wofür sie 30 Minuten braucht.

56. Stefan ist handwerklich sehr begabt.
a. Er geht in die Garage, wo er das Auto repariert.
b. Er geht in die Garage, wo er erst den Toaster, dann die Kaffeemaschine und dann das Auto repariert.
T: Dann beginnt er mit dem Rasenmähen / Ausleihen, wofür er 30 Minuten braucht.
Q: Bringt Stefan das Auto in die Werkstatt?

57. Stefanie interessiert sich für Literatur.
a. Sie geht in die Bibliothek, wo sie ein Gedicht liest.
b. Sie geht in die Bibliothek, wo sie erst ein Sachbuch, dann einen Roman und dann Gedicht liest.
T: Dann beginnt sie mit dem Ausleihen / Beten, wofür sie 10 Minuten braucht.

58. Michael liebt exotisches Essen.
a. Er geht ins Restaurant, wo er den Hummer isst.
b. Er geht ins Restaurant, wo er erst die Garnelen, dann den Krebs und dann den Hummer isst.
T: Dann beginnt er mit dem Trinken / Geldzählen, wofür er 5 Minuten braucht.

59. Dennis will eine Hausapotheke anlegen.
a. Er geht in die Drogerie, wo er ein Medikament heraussucht.
b. Er geht in die Drogerie, wo er erst eine Wundsalbe, dann einen Halstee und dann ein Medikament heraussucht.
T: Dann beginnt er mit dem Bezahlen / Bummeln, wofür er 1 Stunde braucht.

60. Martin hat heute keine Schule.
a. Er geht auf den Spielplatz, wo er mit der Schaufel spielt.
b. Er geht auf den Spielplatz, wo er erst mit den Murmeln, dann dem Fußball und dann mit der Schaufel spielt.

T: Dann beginnt er mit dem <u>Schaukeln</u> / <u>Pauken</u>, wofür er 30 Minuten braucht.
Q: Hat Martin heute schulfrei?

61. Juliana trainiert für den Marathon.
a. Sie geht in den Wald, wo sie eine Ausdauerstrecke läuft.
b. Sie geht in den Wald, wo sie erst eine Aufwärmrunde, dann einen Hopserlauf und dann eine Ausdauerstrecke läuft.
T: Dann beginnt sie mit dem <u>Dehnen</u> / <u>Feiern</u>, wofür sie 3 Stunden braucht.

62. Maike bereitet ein romantisches Abendessen vor.
a. Sie geht ins Esszimmer, wo sie Rosen auf den Tisch stellt.
b. Sie geht ins Esszimmer, wo sie erst den Sektkühler, dann Kerzen und dann Rosen auf den Tisch stellt.
T: Dann beginnt sie mit dem <u>Tischdecken</u> / <u>Einkaufen</u>, wofür sie 30 Minuten braucht.

63. Heinz braucht einen neuen Computer.
a. Er fährt in die Stadt, wo er zu Saturn geht.
b. Er fährt in die Stadt, wo er erst zu Media Markt, dann zu Conrad und dann zu Saturn geht.
T: Dann beginnt er mit dem <u>Vergleichen</u> / <u>Schimpfen</u>, wofür er 10 Minuten braucht.

64. Armin geht in einen Freizeitpark.
a. Er läuft im Park herum, wo er Achterbahn fährt.
b. Er läuft im Park herum, wo er erst Kettenkarussell, dann Autoskooter und dann Achterbahn fährt.
T: Dann beginnt er mit dem <u>Essen</u> / <u>Laufen</u>, wofür er 20 Minuten braucht.
Q: Fährt Armin Riesenrad?

65. Rudolf will Wintersport betreiben.
a. Er geht zur Piste, wo er seine Mütze anzieht.
b. Er geht zur Piste, wo er erst seinen Skianzug, dann seine Stiefel und dann seine Mütze anzieht.
T: Dann beginnt er mit dem <u>Skifahren</u> / <u>Feiern</u>, wofür er 2 Stunden braucht.

66. Sophia fährt in Urlaub.
a. Sie geht zum Kleiderschrank, wo sie ihren Bikini herausholt.
b. Sie geht zum Kleiderschrank, wo sie erst ihren Bikini, dann ihren Sommerrock und dann ihre Flip-Flops herausnimmt.

T: Dann beginnt sie mit dem Packen / Fensterputzen, wofür sie 30 Minuten braucht.

67. Tom ist am Renovieren.
a. Er fährt in den Baumarkt, wo er sich Tapeten kauft.
b. Er fährt in den Baumarkt, wo er sich erst eine Bohrmaschine, dann Gips und dann Tapeten kauft.
T: Dann beginnt er mit dem Tapezieren / Lesen, wofür er 1 Stunde braucht.

68. Angela hat Grippe.
a. Sie geht in die Küche, wo sie sich eine heiße Zitrone macht.
b. Sie geht in die Küche, wo sie sich erst eine Wärmflasche, dann eine Suppe und dann eine heiße Zitrone macht.
T: Dann beginnt sie mit dem Trinken / Recherchieren, wofür sie 30 Minuten braucht.
Q: Ist Angela krank?

69. Conrad hält gleich einen Vortrag.
a. Er geht in den Hörsaal, wo er sein Laptop bereitlegt.
b. Er geht in den Hörsaal, wo er zuerst seine Handouts, dann seinen Zeigestock und dann seinen Laptop bereitlegt.
T: Dann beginnt er mit dem Präsentieren / Verabschieden, wofür er 2 Minuten braucht.

70. Ursula ist am Umziehen.
a. Sie ist im Schlafzimmer, wo sie ihren Kleiderschrank abbaut.
b. Sie ist im Schlafzimmer, wo sie erst ihren Nachttisch, dann ihr Regal und dann ihren Kleiderschrank abbaut.
T: Dann beginnt sie mit dem Einladen /Brunchen, wofür sie 30 Minuten braucht.

71. Maurice spielt Gitarre.
a. Er sitzt auf dem Sofa, wo er ein klassisches Stück spielt.
b. Er sitzt auf dem Sofa, wo er erst eine Ballade, dann einen Popsong und dann ein klassisches Stück spielt.
T: Dann beginnt er mit dem Singen / Fernsehen, wofür er 30 Minuten braucht.

72. Paul ist Bademeister.
a. Er ist im Schwimmbad, wo er zum Sprungturm geht.
b. Er ist im Schwimmbad, wo er erst zum Becken, dann über die Wiese und dann zum Sprungturm geht.
T: Dann beginnt er mit dem Aufpassen / Eincremen, wofür er 30 Minuten braucht.

Q: Ist Paul am Strand?

73. Florian ist sehr gläubig.
a. Er geht in die Kirche, wo er einen Rosenkranz betet.
b. Er geht in die Kirche, wo er erst ein Vater Unser, dann ein Ave Maria und dann einen Rosenkranz betet.

T: Dann beginnt er mit dem Beichten /Prospektlesen, wofür er 30 Minuten braucht.

74. Matthias macht Weihnachtsgeschenke gerne selber.
a. Er geht ins Wohnzimmer, wo er eine Tasche anfertigt.
b. Er geht ins Wohnzimmer, wo er erst Schmuck, dann eine Grußkarte und dann eine Tasche anfertigt.

T: Dann beginnt er mit dem Verpacken / Entrümpeln, wofür er 30 Minuten braucht.

75. Katja hat jetzt Kunstunterricht.
a. Sie geht ins Klassenzimmer, wo sie eine Vase töpfert.
b. Sie geht ins Klassenzimmer, wo sie erst eine Tasse, dann eine Schüssel und dann eine Vase töpfert.

T: Dann beginnt sie mit dem Brennen / Bahnfahren, wofür sie 30 Minuten braucht.

76. Manuel ist durch die Hitze sehr durstig.
a. Er geht in die Küche, wo er das Wasser trinkt.
b. Er geht in die Küche, wo er erst die Cola, dann den Tee und dann das Wasser trinkt.

T: Dann beginnt er mit dem Abwaschen / Zubettgehen, wofür er 30 Minuten braucht.
Q: Ist Manuel warm?

77. Peter will Ordnung auf dem Schreibtisch machen.
a. Er geht ins Büro, wo er die Rechnungen sortiert.
b. Er geht ins Büro, wo er erst die Ordner, dann die Briefe und dann die Rechnungen sortiert.

T: Dann beginnt er mit dem Abheften / Streichen, wofür er 30 Minuten braucht.

78. Tobias ist diese Woche für die Mülltonnen verantwortlich.
a. Er geht in den Hinterhof, wo er den Papiermüll rausstellt.
b. Er geht in den Hinterhof, wo er erst den Gelben Sack, dann den Biomüll und dann den Papiermüll rausstellt.

T: Dann beginnt er mit dem Kehren / Entspannen, wofür er 30 Minuten braucht.

79. Philipp hat einen Amazongutschein bekommen.
a. Er geht ins Internet, wo er sich ein Buch aussucht.
b. Er geht ins Internet, wo er sich erst eine DVD, dann eine CD und dann ein Buch aussucht.
T: Dann beginnt er mit dem Bestellen / Browsen, wofür er 30 Minuten braucht.

80. Lena erwartet heute Gäste zum Mittagessen.
a. Sie geht in die Küche, wo sie die Kartoffeln schält.
b. Sie geht in die Küche, wo sie erst die Karotten, dann die Zwiebeln und dann die Kartoffeln schält.
T: Dann beginnt sie mit dem Kochen / Begrüßen, wofür sie 10 Minuten braucht.
Q: Schält Lena Äpfel?

81. Jennifer will verreisen.
a. Sie geht ins Reisebüro, wo sie sich über einen Flug beraten lässt.
b. Sie geht ins Reisebüro, wo sie sich erst über eine Safari, dann ein Hotel und dann einen Flug beraten lässt.
T: Dann beginnt sie mit dem Buchen / Hausputzen, wofür sie 30 Minuten braucht.

82. Patrick will seine Frau beschenken.
a. Er geht zum Juwelier, wo er sich Uhren anschaut.
b. Er geht zum Juwelier, wo er sich erst Ringe, dann Ketten und dann Uhren anschaut.
T: Dann beginnt er mit dem Auswählen / Heimfahren, wofür er 30 Minuten braucht.

83. Sandra renoviert ihre Wohnung.
a. Sie geht ins Wohnzimmer, wo sie den Boden abdeckt.
b. Sie geht ins Wohnzimmer, wo sie erst die Fußleisten, dann den Türrahmen und dann den Boden abdeckt.
T: Dann beginnt sie mit dem Streichen / Fliesenlegen, wofür sie 30 Minuten braucht.

84. Caroline hat Neuigkeiten.
a. Sie geht zum Telefon, wo sie eine Freundin anruft.
b. Sie geht zum Telefon, wo sie erst einen Kollegen, dann eine Vorgesetzte und dann eine Freundin anruft.
T: Dann beginnt sie mit dem Erzählen / Frühstücken, wofür sie 30 Minuten braucht.
Q: Sendet Caroline die Neuigkeiten per SMS?

85. Sarah packt für eine Dienstreise.

a. Sie geht ins Schlafzimmer, wo sie die Hemden raussucht.
b. Sie geht ins Schlafzimmer, wo sie erst die Röcke, dann die Socken und dann die Hemden raussucht.

T: Dann beginnt sie mit dem <u>Zusammenlegen</u> / <u>Nägelschneiden</u>, wofür sie 10 Minuten braucht.

86. Benjamin hat diese Woche Putzdienst.
a. Er geht ins Badezimmer, wo er die Toilette putzt.
b. Er geht ins Badezimmer, wo er erst die Dusche, dann das Waschbecken und dann die Toilette putzt.

T: Dann beginnt er mit dem <u>Bodenwischen</u> / <u>Prospekteverteilen</u>, wofür er 30 Minuten braucht.

87. Jens hat ein neues Fernglas.
a. Er geht ans Fenster, wo er ein Eichhörnchen beobachtet.
b. Er geht ans Fenster, wo er erst ein Reh, dann einen Vogel und dann ein Eichhörnchen beobachtet.

T: Dann beginnt er mit dem <u>Zeichnen</u> / <u>Polieren</u>, wofür er 10 Minuten braucht.

88. Thomas ist Pfleger im Zoo.
a. Er geht zu den Gehegen, wo er die Hasen beobachtet.
b. Er geht zu den Gehegen, wo er erst die Zebras, dann die Affen und dann die Hasen beobachtet.

T: Dann beginnt er mit dem <u>Füttern</u> / <u>Kinderbelustigen</u>, wofür er 30 Minuten braucht.

Q: Ist Thomas Tierpfleger?

89. Susanne schreibt eine Arbeit über Insekten.
a. Sie geht in den Wald, wo sie die Ameisen untersucht.
b. Sie geht in den Wald, wo sie erst die Käfer, dann die Blattläuse und dann die Ameisen untersucht.

T: Dann beginnt sie mit dem <u>Dokumentieren</u> / <u>Händewaschen</u>, wofür sie 2 Minuten braucht.

90. Markus ist ein pflichtbewusster Bürger.
a. Er geht auf die vereiste Straße, wo er die Einfahrt räumt.
b. Er geht auf die vereiste Straße, wo er erst den Gartenpfad, dann den Bürgersteig und dann die Einfahrt räumt.

T: Dann beginnt er mit dem <u>Salzstreuen</u> / <u>Schneemannbauen</u>, wofür er 30 Minuten braucht.

91. Karin bereitet sich auf Silvester vor.
a. Sie fährt ins Einkaufszentrum, wo sie Raketen kauft.
b. Sie fährt ins Einkaufszentrum, wo sie erst Konfetti, dann Böller und dann Raketen kauft.
T: Dann beginnt sie mit dem <u>Knallen</u> / <u>Schlendern</u>, wofür sie 30 Minuten braucht.

92. David braucht noch Zutaten für die Pasta.
a. Er geht ins Geschäft, wo er die Tomaten kauft.
b. Er geht ins Geschäft, wo er erst die Nudeln, dann das Hackfleisch und dann die Tomaten kauft.
T: Dann beginnt er mit dem <u>Zubereiten</u> / <u>Filmschauen</u>, wofür er 2 Stunden braucht.
Q: Kauft Thomas Zucchini?

93. Claudia hat ihre Uhr verlegt.
a. Sie geht ins Schlafzimmer, wo sie die Schublade durchsucht.
b. Sie geht ins Schlafzimmer, wo sie erst erst die Truhe, dann den Nachttisch und dann die Schublade durchsucht.
T: Dann beginnt sie mit dem <u>Aufräumen</u> / <u>Aufgabenmachen</u>, wofür sie 30 Minuten braucht.

94. Anna ist zu einem Geburtstag eingeladen.
a. Sie geht in die Küche, wo sie einen Kuchen backt.
b. Sie geht in die Küche, wo sie erst eine Torte, dann ein Brot und dann einen Kuchen backt.
T: Dann beginnt sie mit dem <u>Verzieren</u> / <u>Schminken</u>, wofür sie 30 Minuten braucht.

95. Robert muss sich auf ein wichtiges Spiel vorbereiten.
a. Er geht auf den Tennisplatz, wo er den Aufschlag trainiert.
b. Er geht auf den Tennisplatz, wo er erst die Rückhand, dann das Linienspiel und dann den Aufschlag trainiert.
T: Dann beginnt er mit dem <u>Laufen</u> / <u>Telefonieren</u>, wofür er 5 Minuten braucht.

96. Katharina hat gestern eine Party gegeben.
a. Sie geht durch die Wohnung, wo sie den Flur aufräumt.
b. Sie geht durch die Wohnung, wo sie erst die Küche, dann das Bad und dann den Flur aufräumt.
T: Dann beginnt sie mit dem <u>Lüften</u> / <u>Brötchenholen</u>, wofür sie 30 Minuten braucht.
Q: Hat Katharina gestern gefeiert?

97. Felix hat den Tanzkurs fertig.
a. Er geht auf den Abschlussball, wo er einen Foxtrott tanzt.
b. Er geht auf den Abschlussball, wo er erst einen Walzer, dann einen Cha-Cha-Cha und dann einen Foxtrott tanzt.
T: Dann beginnt er mit dem Feiern / Abendessen, wofür er 30 Minuten braucht.

98. Maria macht ihren Führerschein.
a. Sie geht auf den Verkehrsübungsplatz, wo sie das Anfahren übt.
b. Sie geht auf den Verkehrsübungsplatz, wo sie erst das Bremsen, dann das Einparken und dann das Anfahren übt.
T: Dann beginnt sie mit dem Rundendrehen / Fensterwischen, wofür sie 30 Minuten braucht.

99. Oliver bereitet das Osterfest vor.
a. Er geht in die Küche, wo er Eier holt.
b. Er geht in die Küche, wo er erst Schokolade, dann Kuchen und dann Eier holt.
T: Dann beginnt er mit dem Färben / Beten, wofür er 10 Minuten braucht.

100. Melanie ist Lehrerin in der Grundschule.
a. Sie geht ins Lehrerzimmer, wo sie die Klassenarbeit korrigiert.
b. Sie geht ins Lehrerzimmer, wo sie erst einen Aufsatz, dann die Hausaufgaben und dann die Klassenarbeit korrigiert.
T: Dann beginnt sie mit dem Benoten / Rauchen, wofür sie 5 Minuten braucht.
Q: Korrigiert Melanie die Arbeiten zu Hause?

101. Nils liebt Beethoven.
a. Er geht zu seiner CD-Sammlung, wo er eine Sinfonie raussucht.
b. Er geht zu seiner CD-Sammlung, wo er erst ein Konzert, dann eine Oper und dann eine Sinfonie raussucht.
T: Dann beginnt er mit dem Hören / Bodenwischen, wofür er 30 Minuten braucht.

102. Johannes ist auf einer Weinprobe.
a. Er geht zum Probiertisch, wo er einen Rotwein verköstigt.
b. Er geht zum Probiertisch, wo er erst einen Sekt, dann einen Rosé und dann einen Rotwein verköstigt.
T: Dann beginnt er mit dem Bewerten / Lesen, wofür er 1 Stunde braucht.

103. Kathrin kauft auf dem Markt ein.
a. Sie geht zu den Ständen, wo sie einen Fisch aussucht.

b. Sie geht zu den Ständen, wo sie erst eine Gurke, dann einen Käse und dann einen Fisch aussucht.

T: Dann beginnt sie mit dem <u>Bezahlen</u> / <u>Fahrradfahren</u>, wofür sie 30 Minuten braucht.

104. Annika redet sonntags gerne mit ihren Verwandten.

a. Sie geht ans Telefon, wo sie die Nummer ihrer Tante raussucht.

b. Sie geht ans Handy, wo sie die erst Nummer einer Nichte, dann die eines Cousins und dann die einer Tante raussucht.

T: Dann beginnt sie mit dem <u>Telefonieren</u> / <u>Teppichklopfen</u>, wofür sie 30 Minuten braucht.

Q: Sucht sie die Nummer ihres Onkels heraus?

105. Simon macht Urlaub in Paris.

a. Er geht durch die Innenstadt, wo er den Eiffelturm besucht.

b. Er geht durch die Innenstadt, wo er erst Notre Dame, dann den Eiffelturm und dann den Louvre besucht.

T: Dann beginnt er mit dem <u>Fotografieren</u> / <u>Abreisen</u>, wofür er 30 Minuten braucht.

106. Tanja macht ihre Steuererklärung.

a. Sie geht an den Schreibtisch, wo sie die Adresse hinschreibt.

b. Sie geht an den Schreibtisch, wo sie erst den Arbeitgeber, dann die Steuernummer und dann ihre Adresse hinschreibt.

T: Dann beginnt sie mit dem <u>Ausfüllen</u> / <u>Musizieren</u>, wofür sie 30 Minuten braucht.

107. Nina braucht Fahrkarten für sich und ihre Kinder.

a. Sie geht zum Automaten, wo sie ein Mehrfachticket kauft.

b. Sie geht zum Automaten, wo sie erst eine Kurzstreckenkarte, dann eine Monatskarte und dann ein Mehrfachticket kauft.

T: Dann beginnt sie mit dem <u>Entwerten</u> / <u>Getränkekaufen</u>, wofür sie 2 Minuten braucht.

108. Nicole muss viel für die Schule machen.

a. Sie geht in ihr Zimmer, wo sie eine Bruchrechnung bearbeitet.

b. Sie geht in ihr Zimmer, wo sie erst einen Aufsatz, dann eine Pflanzenbeschreibung und dann eine Bruchrechnung bearbeitet.

T: Dann beginnt sie mit dem <u>Addieren</u> / <u>Reiten</u>, wofür sie 30 Minuten braucht.

Q: Hat Nicole viele Hausaufgaben?

109. Sabrina ist karnevalsbegeistert.

a. Sie stellt sich vor den Spiegel, wo sie sich ein Hexenkostüm vorhält.
b. Sie stellt sich vor den Spiegel, wo sie sich erst ein Piraten-, dann ein Clowns-, und dann ein Hexenkostüm vorhält.

T: Dann beginnt sie mit dem Anprobieren / Tanzen, wofür er sie Minuten braucht.

110. Johanna rätselt gerne.

a. Sie setzt sich aufs Sofa, wo sie ein Sudoku herausholt.
b. Sie setzt sich aufs Sofa, wo sie erst eine Logelei, dann ein Kreuzworträtsel und dann ein Sudoku herausholt.

T: Dann beginnt sie mit dem Lösen / Teetrinken, wofür sie 30 Minuten braucht.

111. Fabian will seinen Vater ärgern.

a. Er geht an den Werkzeugkasten, wo er die Schrauben raussucht.
b. Er geht an den Werkzeugkasten, wo er erst die Bohreraufsätze, dann die Muttern und dann die Schrauben raussucht.

T: Dann beginnt er mit dem Verstecken / Staubsaugen, wofür er 30 Minuten braucht.

112. Laura will für Halloween schmücken.

a. Sie geht in die Abstellkammer, wo sie den Kürbis holt.
b. Sie geht in die Abstellkammer, wo sie erst die Gummischlangen, dann die Papierfledermäuse und dann den Kürbis holt.

T: Dann beginnt sie mit dem Aushöhlen / Schminken, wofür sie 10 Minuten braucht.
Q: Holt sie die Dekoration vom Speicher?

113. Dominik muss sein Fahrrad instand setzen.

a. Er geht in die Garage, wo er das Fahrradlicht repariert.
b. Er geht in die Garage, wo er erst die Pedale, dann die Bremse und dann das Fahrradlicht repariert.

T: Dann beginnt er mit dem Fahren / Wandstreichen, wofür er 30 Minuten braucht.

114. Christina will abschalten.

a. Sie geht ins Wohnzimmer, wo sie eine Talkshow anschaut.
b. Sie geht ins Wohnzimmer, wo sie erst eine Sitcom, dann einen Spielfilm und dann eine Talkshow anschaut.

T: Dann beginnt sie mit dem Einschlafen / Epilieren, wofür sie 5 Minuten braucht.

115. Lars spielt dem Kollegen einen Streich.

a. Er geht ins Büro, wo er die Tastatur versteckt.

b. Er geht ins Büro, wo er erst den Mülleimer, dann den Füller und dann die Tastatur versteckt.
T: Dann beginnt er mit dem <u>Arbeiten</u> / <u>Spazierengehen</u>, wofür er 6 Stunden braucht.

116. Daniela geht heute aus.
a. Sie geht ins Bad, wo sie sich die Augen schminkt.
b. Sie geht ins Bad, wo sie sich erst die Lippen, dann die Wangen und dann die Augen schminkt.
T: Dann beginnt sie mit dem <u>Frisieren</u> / <u>Aufzugfahren</u>, wofür er 30 Minuten braucht.
Q: Schminkt Daniela sich im Bad?

117. Julian sprüht nachts Graffittis.
a. Er geht in die Stadt, wo er eine Wand besprüht.
b. Er geht in die Stadt, wo er erst ein Schaufenster, dann ein Baugerüst und dann eine Wand besprüht.
T: Dann beginnt er mit dem <u>Wegrennen</u> / <u>Spazierengehen</u>, wofür er 30 Minuten braucht.

118. Juliane will eine Teeparty machen.
a. Sie geht in ihr Kinderzimmer, wo sie ihren Teddybären an den Tisch setzt.
b. Sie geht in ihr Kinderzimmer, wo sie erst ihre Puppe, dann ihren Stoffhasen und dann ihren Teddybären an den Tisch setzt.
T: Dann beginnt sie mit dem <u>Einschenken</u> / <u>Weinen</u>, wofür sie 10 Minuten braucht.

119. Tim ist mit dem Essen fertig.
a. Er geht zum Waschbecken, wo er den Teller spült.
b. Er geht zum Waschbecken, wo er erst das Besteck, dann das Glas und dann den Teller spült.
T: Dann beginnt er mit dem <u>Abtrocknen</u> / <u>Wäschebügeln</u>, wofür er 30 Minuten braucht.

120. Sonja gibt einen Sektempfang.
a. Sie geht durch den Raum, wo sie einen Freund begrüßt.
b. Sie geht durch den Raum, wo sie erst einen Bekannten, dann einen Verwandten und dann einen Freund begrüßt.
T: Dann beginnt sie mit dem <u>Ausschenken</u> / <u>Aufsatzschreiben</u>, wofür sie 10 Minuten braucht.
Q: Hat Sonja Gäste?
